# *De Novo* Transcriptome Analysis Provides Insights into Immune Related Genes and the RIG-I-Like Receptor Signaling Pathway in the Freshwater Planarian (*Dugesia japonica*)

**DOI:** 10.1371/journal.pone.0151597

**Published:** 2016-03-17

**Authors:** Qiuxiang Pang, Lili Gao, Wenjing Hu, Yang An, Hongkuan Deng, Yichao Zhang, Xiaowen Sun, Guangzhong Zhu, Baohua Liu, Bosheng Zhao

**Affiliations:** 1 Laboratory of Developmental and Evolutionary Biology, School of Life Sciences, Shandong University of Technology, Zibo 255049, China; 2 Anti-aging & Regenerative Medicine Research Institution, School of Life Sciences, Shandong University of Technology, Zibo 255049, China; 3 Immolife-biotech Co., Ltd., Nanjing 210000, China; 4 Shenzhen University Health Science Center, Shenzhen 518060, China; University of Lausanne, SWITZERLAND

## Abstract

**Background:**

The freshwater planarian *Dugesia japonica* (*D*. *japonica*) possesses extraordinary ability to regenerate lost organs or body parts. Interestingly, in the process of regeneration, there is little wound infection, suggesting that *D*. *japonica* has a formidable innate immune system. The importance of immune system prompted us to search for immune-related genes and RIG-I-like receptor signaling pathways.

**Results:**

Transcriptome sequencing of *D*. *japonica* was performed on an IlluminaHiSeq2000 platform. A total of 27,180 transcripts were obtained by Trinity assembler. CEGMA analysis and mapping of all trimmed reads back to the assembly result showed that our transcriptome assembly covered most of the whole transcriptome. 23,888 out of 27,180 transcripts contained ORF (open reading fragment), and were highly similar to those in *Schistosoma mansoni* using BLASTX analysis. 8,079 transcripts (29.7%) and 8,668 (31.9%) were annotated by Blast2GO and KEGG respectively. A *DYNLRB-like* gene was cloned to verify its roles in the immune response. Finally, the expression patterns of 4 genes (*RIG-I*, *TRAF3*, *TRAF6*, *P38*) in the RIG-I-like receptor signaling pathway were detected, and the results showed they are very likely to be involved in planarian immune response.

**Conclusion:**

RNA-Seq analysis based on the next-generation sequencing technology was an efficient approach to discover critical genes and to understand their corresponding biological functions. Through GO and KEGG analysis, several critical and conserved signaling pathways and genes related to RIG-I-like receptor signaling pathway were identified. Four candidate genes were selected to identify their expression dynamics in the process of pathogen stimulation. These annotated transcripts of *D*. *japonica* provide a useful resource for subsequent investigation of other important pathways.

## Introduction

The freshwater planarian *Dugesia japonica* (*D*. *japonica*) is a remarkable creature with extraordinary regenerative abilities. They have been used as model animals for years for study of biological evolution processes and occupy a key position in the field of animal phylogeny [1]. In recent years, researchers have shown an increased interest in *D*. *japonica*, not only because they can regenerate entire triploblastic body plan from small fragments [2], but also they can continuously renew all cell types from pluripotent stem cells (neoblasts) [3,4]. The increasingly unraveled regenerative capacity of planarian, which is not possessed by other commonly used invertebrate model, makes it a valuable system for studying regeneration and development [5]. It is noteworthy that there is little wound infection in regenerated planarians, suggesting that planarians may possess a complicated and effective immune system [6,7]. Intermingling planarian wound response and regeneration will provide an exciting opportunity to advance our knowledge of repair mechanism and immune defense system.

Planarian does not have an adaptive immune system (specificity); instead, it has developed innate immunity (non-specificity) as the dominant system of biological host defense [8–10]. As the first line of defense, the surface structures and tissues in gastrointestinal tract form a physical barrier for invading pathogens and are essential for the animal. Beyond that, the most important mechanism is the recognition of infectious non-self. Studies shown that a number of proteins, such as pattern recognition receptors (PRRs), recognize common antigens existed on the surface (pathogen associated molecular patterns, PAMPs) of pathogens according to the pattern recognition to resist and expel foreign substances [11,12]. Subsequently, non-self recognition signals trigger a series of cascade reactions through signal modulation and amplification and activate corresponding signal transduction pathways [13,14].

In invertebrates, eight groups of PRRs have been reported, including peptidoglycan recognition proteins (PGRPs), thioester-containing proteins (TEPs), Gram-negative binding proteins (GNBPs), scavenger receptors (SCRs), C-type lectin (CTL), galectins (GALEs), toll-like receptor (TLR) and fibrinogen-like domain immune lectins (FBNs) [15–17]. These proteins are indispensable for immune responses including phagocytosis, coating and nodule [18], which are activated through protease cascade reaction in multifold signaling pathways. Thus far, in invertebrates, a great many studies of immune related signaling pathways within *Drosophila melanogaster* have been reported, including Toll-like, IMD and JAK/STAT signaling pathway [19–21]. The same and other pathways also exist in other invertebrates, for example, DBL, DAF-2/DAF-16, MAPK, Toll-like pathways in *Caenorhabditis elegans* [22–25], Toll-like and IMD pathways in crustaceans [26,27]. However, antiviral innate immune signal transduction pathways mediated by retinoic acid inducible-gene I (RIG-I)-like receptor and nucleotide-binding oligomerization domain-containing protein (NOD)-like receptor signaling pathway have not been reported in invertebrates.

RIG-I is a member of pattern recognition receptors (PRRs) and plays a pivotal role in immune response by recognizing and binding the double stranded RNAs and 5'-triphosphate single stranded RNAs of invading virus [28,29]. After binding with virus nucleic acid, RIG-I forms a complex with an adaptor protein MAVs/VISA/Cardif/IPS-1 which is anchored on the outer mitochondrial membrane [30–33]. Then, the complex on the membrane recruits tumor necrosis factor receptor associated factor 3 (TRAF3) and TRAF6 through the TIM binding sites on MAVs and activates related transcription factors, including nuclear factor-κB (NF-κB), interferon-regulated-factor (IRF) and type I interferons (IFN-α/β) [30,34]. TRAFs regulate cell physiological and pathological processes through multiple signaling pathways and participate in immune response [35]. Reports have showed that the activation of IFN-α/β in RIG-I-like receptor signaling pathway requires the participation of P38 [36,37]. The activity of P38 is essential for viral elimination of IFN-α/β.

The importance of innate immunity in invertebrates is indisputable, while the pivotal immune-related genes and signaling pathways are poorly understood in *D*. *japonica*. The emergence of high-throughput sequencing technologies has permitted new approaches and designs in investigation of functional genes involved in various specific signal-transduction and metabolic and pathways. *De novo* transcriptome analysis has been employed as an appropriate technique for identifying unknown genes in non-model organisms [38].

Here, we sequenced the transcriptome of *D*. *japonica* (clonal strain BS, called Dj-BS) using IlluminaHiSeq2000, and assembled the transcriptome using Trinity (http://trinityrnaseq.sourceforge.net/) after quality filtration and trimming of raw reads. ORF prediction, functional annotation, GO (Gene Ontology) analysis, and KEGG (Kyoto encyclopedia of genes and genomes database) analysis were performed. Immune-related genes and immune system related pathways were also identified and the expression patterns of four candidate genes involved in RIG-I-like receptor signaling pathway were identified after stimulation with lipopolysaccharide (LPS) and peptidoglycan (PGN). This study will make an important contribution to the understanding of planarian innate immune system, especially in discovering those immune-related genes in planarian.

## Results and Discussion

### Sequencing and *de novo* assembly

**Fig 1:**
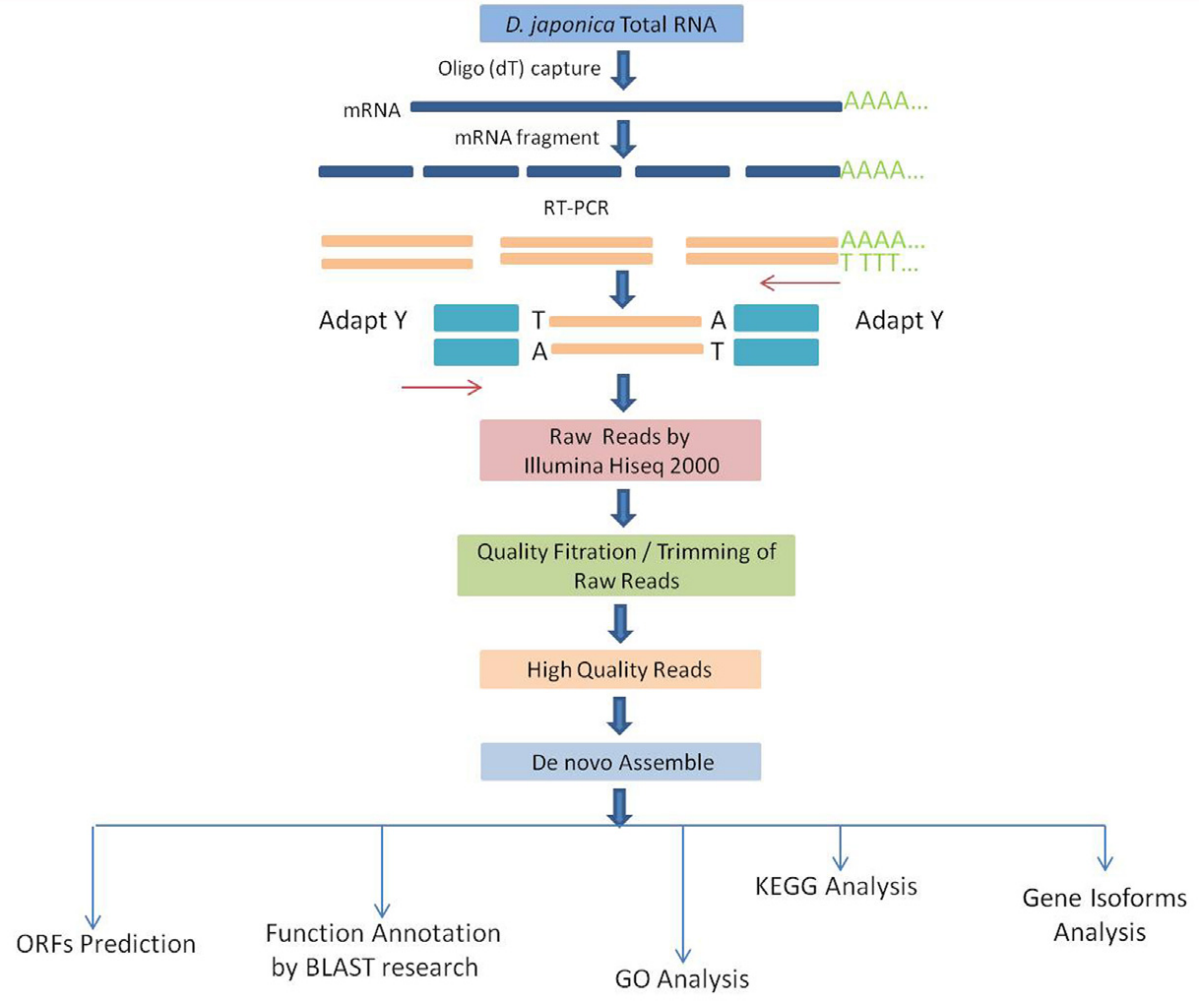
Workflow of Dj-BS transcriptome project.

Workflow for cDNA preparation, sequencing, assembly, and annotation of Dj-BS transcriptome is presented in **Fig 1**. cDNA libraries were constructed from Dj-BS mRNA and were sequenced using an IlluminaHiSeq2000 sequencing platform. Original images were translated into sequences by base calling, and a total of 42,877,438 paired-end raw reads were obtained. The sequences account for approximately 4.3G bp with a Q20 (proportion of nucleotides with quality value larger than 20 in reads) over 92.87% and numerical value of N% is very low (S1 File). Low quality reads, which contain adaptors, many Ns and low quality scores, and short reads (<20 bp in length) were removed. In total, 40,449,653 high quality reads with an average length of 90 bp were generated. Approximately, 95% filtered reads were obtained and used for future analysis.

High quality reads were assembled using the Trinity program (http://trinityrnaseq.sourceforge.net/) [39]. A total of 27,180 transcripts (including all isoforms from alternative splicing) were obtained, with an average length of 958 nt and N50 length of 1,196 nt, which consist in 21,536 genes **(Table 1)**. Among these, there were 12,119 transcripts (44.59%) with a length between 400 to 800 nt, and the length of longest and shortest was 12,141 and 351 nt, respectively (S2 File).

**Table 1:** Summary of sequencing and assembly of Dj-BS transcriptome.

Item	Value
Number of raw reads	42,877,438
Number of filtered reads	40,449,653
Average length of filtered reads (nt)	90
Number of assembled transcripts	27,180
Size of all transcripts (Mbp)	26
Average length of transcripts (nt)	958
N50 of transcripts (nt)	1,196
Longest transcript (nt)	12,141
Number of genes	21,536

### Assessment of assembly

To determine the integrity of the transcriptome assembly, the completeness of our transcriptome assembly was assessed by using CEGMA and by mapping of all trimmed reads back to the assembly result. 218 out of the 248 core proteins (87.9%) were defined as ‘complete’ by CEGMA, and 89% of all trimmed pair-end reads were mapped back to the final assembled transcriptome by Bowtie2. These results indicated that our transcriptome assembly covered most of the whole Dj-BS transcriptome. Another parameter for assessing the quality of a transcriptome assembly is the number of assembled transcripts that appear to be full-length. To perform the full-length transcript analysis, we aligned the assembled transcripts against all proteins from Uniprot protein database with to find unique top matching proteins, and then calculated the percentage of the protein length that covered by our assembled transcripts. Our results showed that the top matching proteins, which had ≥80% of their sequences covered by the assembled transcripts, occupied 51% (3,747/7,279) of all matched proteins **(Table 2)**.

**Table 2:** BlastX VS Uniprot protein database.

Coverage rate (%)	Protein count	Total count
100	2,080	2,080
90	979	3,059
80	688	3,747
70	527	4,274
60	511	4,785
50	538	5,323
40	629	5,952
30	646	6,598
20	494	7,092
10	187	7,279

Although the final transcript number of the Dj-BS transcriptome assembly (27,180) is 26.9% less than the *D*. *japonica* transcriptome in Qin *et al*’s work (37,218, named Dj-Qin trancriptome in this paper for comparison) [40], the average length of our assembled transcriptome (958 nt) is twice as long as the average length of their transcriptome (468 nt), suggesting that our assembled Dj-BS transcriptome is more complete. Compared with the 1,456 up-regulated transcripts from the work by Abnave *et al* [6], all could find the corresponding hits in our transcriptome by TBLASTX, and 918 of those are highly conserved sequences in our transcriptome showed by the BLASTN result (E-value ≤ 1e-5). These results showed that the assembly quality was sufficiently high for the subsequent functional analysis.

### Functional annotation of assembled transcripts

**Fig 2:**
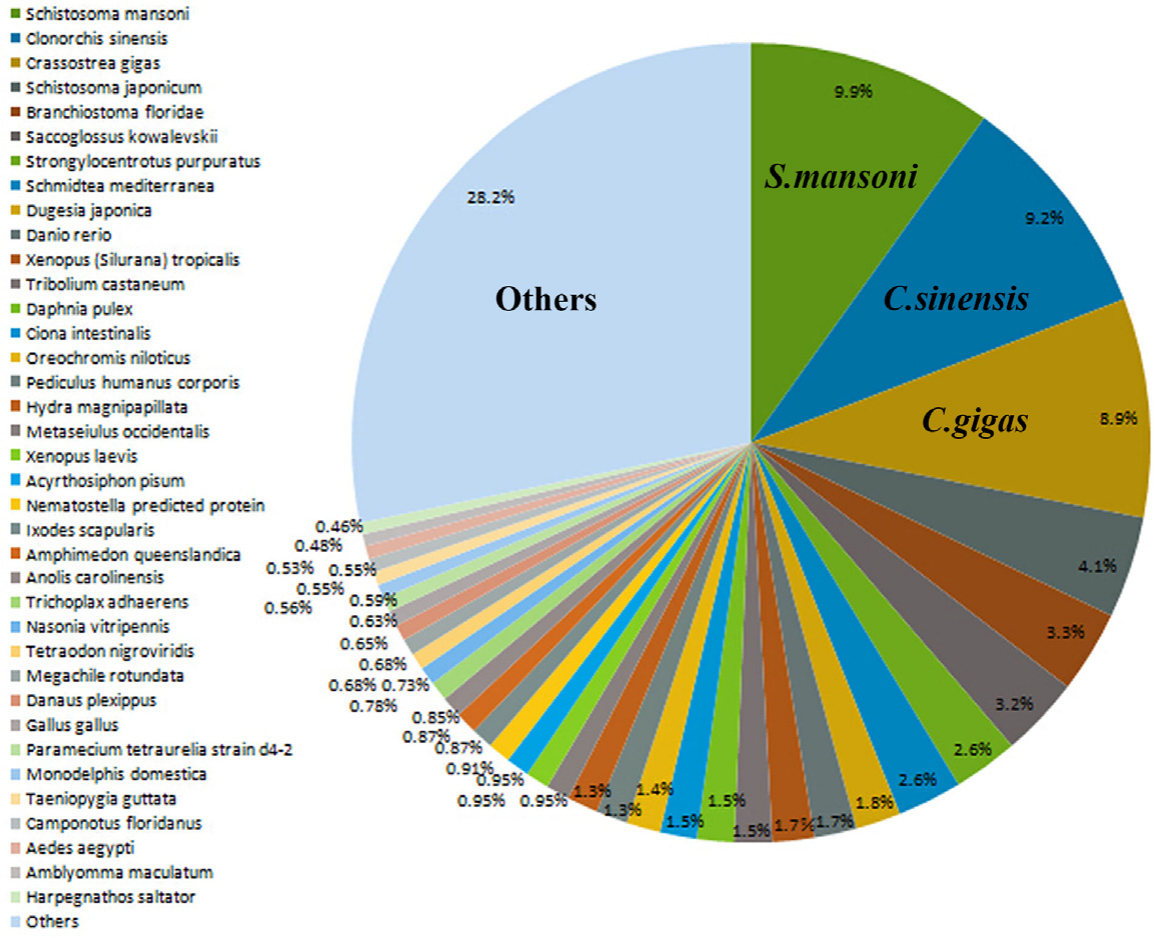
BLASTX Top-Hit species distribution of transcripts.

The ORF prediction of assembled transcripts was carried out using Trinity. The results showed that 23,888 out of 27,180 transcripts contained ORF candidates. After those ORFs were converted into proteins, functional annotation was performed on the NCBI website by BLASTP [41] with the NCBINR, STRING and GENE database. 11,140 and 1,504 proteins were found from the protein databases NCBINR and STRING, respectively. Then, all assembled sequences were aligned with sequences from the NR, STRING and GENE database by BLASTX (BLAST Version 2.2.25, E-value ≤ 1e-5). We discovered that these transcripts in Dj-BS showed significant similarity with those in *Schistosoma mansoni* (9.9%), followed by those in *Clonorchis sinensis* (9.2%) and *Crassostra gigas* (8.9%) **(Fig 2 and Table 3)**.

**Table 3:** All-in-one list of annotations.

**Total transcript**	27,180	100%
**NR, STRING, GENE**	11,103	40.8%
**Uniprot**	7,279	30.1%
**KEGG**	8,668	31.9%
**GO**	8,079	29.7%

### GO annotation

**Fig 3:**
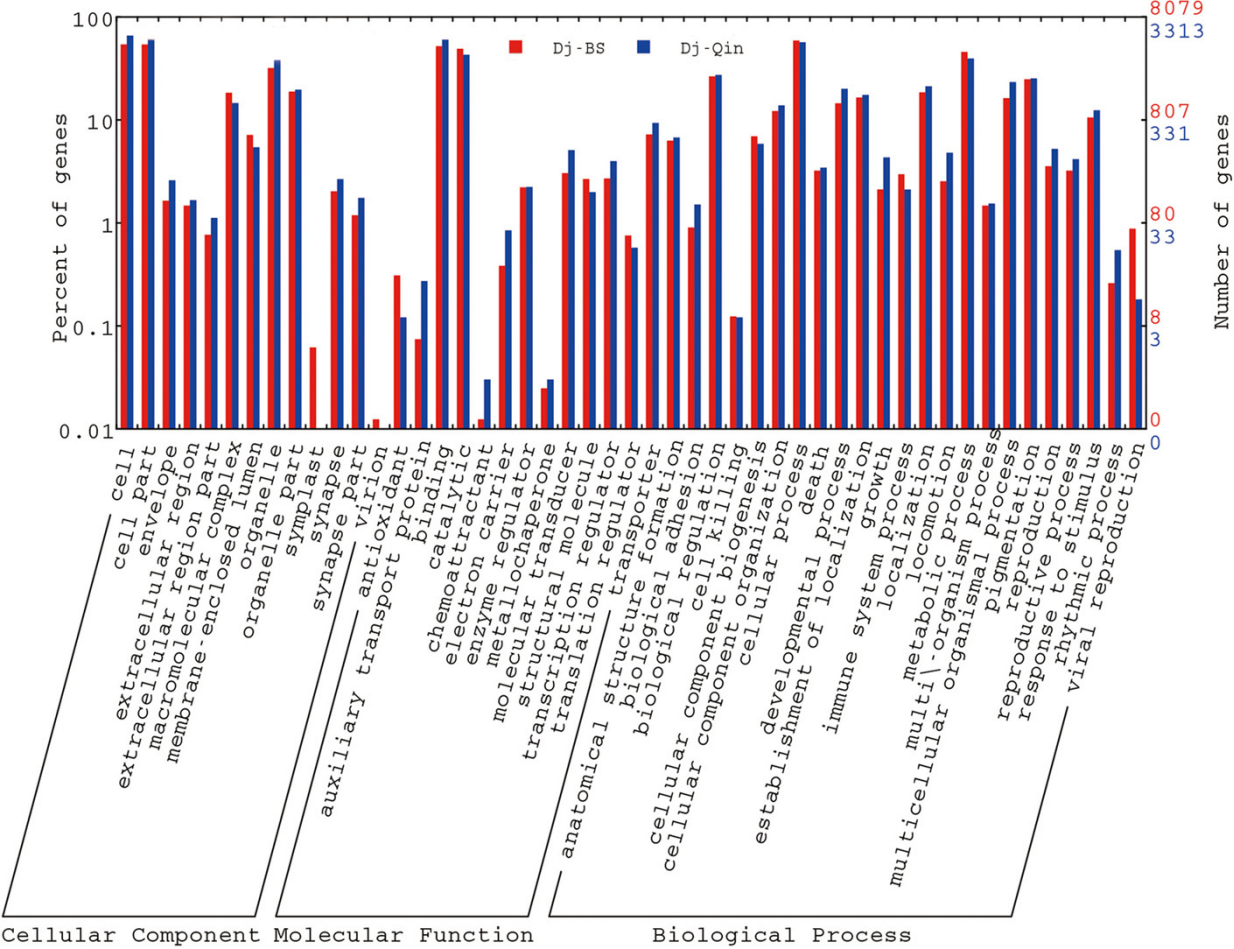
GO functional classification of transcripts in Dj-BS and Dj-Qin.

Gene Ontology (GO) assignments [42] were used to classify the functions of transcripts, which help us understand the biological significance of the tested genes. 8,079 of Dj-BS transcripts (29.7%) were annotated by Blast2GO (http://www.blast2go.com/b2ghome) **(Table 3)**, and gene functional classification was plotted by WEGO (http://wego.genomics.org.cn/) (**Fig 3**). These annotated transcripts can be classified into three categories in GO assignments: cellular component (GO: 00005575), molecular function (GO: 0003674) and biological process (GO: 0008150). Under the cellular component category, which contains 13 functional classes, classes for cell and cell part account for the major proportion with 4,370 (54.1%) and 4,368 (54.07%) transcripts, respectively. Under the molecular function category, which contains 14 functional classes, classes for binding and catalytic activity accounted for the preponderant portion, with 4,198 (51.96%) and 3,970 (49.14%) transcripts, respectively. In the biological process category, which contains 24 functional classes, classes for cellular process and metabolic process were dominant, with cellular process 4,772 (59.07%) and metabolic process 3,699 (45.79%) transcripts, respectively. In addition, 240 (2.97%) transcripts were assigned to the class for immune system process (S3 and S4 Files). To further analyze the GO annotation results, we compared the GO annotation between our Dj-BS transcriptome to the previous Dj-Qin transcriptome (**Fig 3** and S5 File). Because the transcripts might belong to the same species, it is reasonable that, as a percentage of classified transcripts, the predicted transcript sets for Dj-BS and Dj-Qin are very similarly distributed among different functional categories. In addition, in almost all functional categories, the number of annotated from Dj-BS is much bigger than the number in Dj-Qin, and the total annotated transcripts (8,079) from Dj-BS are twice more than those from Dj-Qin (3,313) (S5 File), which indicated Dj-BS transcriptome provides a better platform for future study of planarian *D*. *japonica*.

### Functional classification using KEGG

Spatial and temporal regulations are essential events for life. In recent years, investigation of relationships among proteins, in each specific pathway, has received considerable attention. KEGG [43] is a large knowledge base, which connects the genomic information and functional information and provides large-scale analyses of biological functions in a systematic manner. In this study, by BLASTX, we discovered 3,511 sequences from KEGG GENE database belonging to 300 pathways, which matched 8,668 transcripts in our results. Among these, 507 transcripts (396 genes) matched with 150 orthologues, which are involved in 15 KEGG immune-related pathways (S4 and S6 Files). Combining with the KEGG and GO annotation results, we were able to identify 240 immune-related genes involved in 165 KEGG pathways from Dj-BS. Conserved pathways were found both in planarians and higher animals, such as complement and coagulation cascades, RIG-I-like receptor, Toll-like receptor, NOD-like receptor and FcεRI signaling pathway.

### Dj-BS immune associated genes

As a platyhelminthes, inside the planarian there must be a mechanism which prevent and defend the invasion and infection of pathogens. However, the planarian immune system is still largely unknown [44]. We selected genes in 15 KEGG immune-related pathways firstly. Genes participated in the immune response which have been previously verified are presented in **Table 4** and S7 File. All these sequences were aligned with sequences from NCBI to find conserved domains. These candidate genes include PRRs (C-lectin, A2M, RIG-I), crucial factors participated in multiple signaling pathways (PI3K, P38, JNK, MAPK, AKT), TRAF family proteins, tolloid-like proteins, tyrosinase, CUTA-like protein and DYNLRB-like protein. C-type lectin, as an emblematic PRR, mediated a variety of immune responses within invertebrates, such as agglutinating bacteria, identifying specific sugar and promoting effect on phagocytosis [45,46]. Tyrosinase, also known as phenoloxidase, is an important element of the immune reaction process resisted pathogens or parasites in invertebrates [47–49]. Importantly, Abnave *et al* found that *CUTA-like* and *DYNLRB-like* genes actively contribute to the elimination of *L*. *pneumophila* and *S*. *aureus* (bacteria which were selected to stimulate planarians for producing immune response) in planarian *D*. *japonica* [6]. Therefore, detection of candidate genes and further functional investigation will advance the understanding of planarian immune system.

**Table 4:** Potential candidate genes of Dj-BS innate immune system.

Innate immune component	Domain found	Query name from Dj-BS transcriptome	Species with top matching	Percentage identity
C-lectin	C-type lectin like domain	comp3250_c0_seq1	*Girardia tigrina*	76%
Mannan binding lectin (MBL)	C-type lectin like domain	comp11867_c0_seq1	*Girardia tigrina*	56%
Alpha 2-macroglobulin	A2M domain	comp5655_c0_seq3	*Meleagris gallopavo*	56%
Tumornecrosis factor receptor associated factor 1 (TRAF1)	TRAF-type zinc finger + MATH domain	comp12242_c0_seq1	*Taeniopy giaguttata*	64%
TRAF2	TRAF-type zinc finger + MATH domain	comp4820_c0_seq1	*Dugesia japonica*	98%
TRAF3	TRAF-type zinc finger + MATH domain	comp4190_c0_seq1	*Crassostrea gigas*	64%
TRAF4	TRAF-type zinc finger + MATH domain	comp13793_c0_seq1	*Amphimedon queenslandica*	54%
TRAF5	TRAF-type zinc finger + MATH domain	comp10759_c0_seq1	*Saccoglossus kowalevskii*	57%
TRAF6	TRAF-type zinc finger + MATH domain	comp14413_c0_seq1	*Schmidtea mediterranea*	84%
TRAF7	TRAF-type zinc finger + MATH domain	comp19328_c0_seq1	*Ciona intestinalis*	60%
Tolloid-like	ZnMc domain + CUB domain	comp18577_c0_seq1	*Schmidtea mediterranea*	74%
Toll-interacting protein (TOLLIP)	C2 domain + CUE domain	comp1933_c0_seq1	*Oikopleura dioica*	64%
Retinoic acid inducible gene (RIG-I)	RLR-like + HELIC domain + DEXD domain	comp12120_c0_seq2	*Crassostrea gigas*	68%
P38	Catalytic domain + Serine/Threonine Kinase	comp797_c0_seq1	*Clonorchis sinensis*	76%
LPS-induced TNF-alpha factor	LITAF-like zinc ribbon domain	comp2752_c0_seq3	*Solen grandis*	89%
pi3k-like protein	PI3K domain	comp24438_c0_seq1	*Schmidtea mediterranea*	89%
AKT	PH_PKB domain + STK_PKB domain	comp3600_c0_seq1	*Schistosoma mansoni*	82%
Mitogen-activated protein kinase (MAPK)	PB1 domain + Catalytic domain + Serine/Threonine Kinase	comp8915_c0_seq1	*Danio rerio*	58%
Tyrosine-protein kinase	Tyrosinase domain	comp12726_c0_seq1	*Clonorchis sinensis*	65%
Urokinase plasminogen activator (PLAU)	Kringle domain + Tryp_SP domain	comp846_c0_seq1	*Dugesia japonica*	68%
DYNLRB-like	Roadblock/LC7 domain	comp3021_c0_seq1	*Saccoglossus kowalevskii*	86%
CUTA-like	CutA1 domain	comp659_c0_seq1	*Oryza sativa Japonica Group*	72%
Protein inhibitor of activated STAT2 (PIAS2)	PINIT domain + MIZ/SP-RING zinc finger	comp12694_c0_seq1	*Pediculus humanus corporis*	63%
c-Jun N-terminal Kinase (JNK)	STKc_JNK domain	comp2310_c0_seq1	*Schistosoma mansoni*	87%

LPS and PGN are commonly used as a wide range of immune stress reagents for stimulating invertebrates to produce immune response [50,51], and in this study we also used LPS and PGN in planarian immune respons research. To verify genes participating in the immune response and to confirm the stimulation of LPS and PGN can cause the immune response of planarians, we cloned the *DYNLRB-like* gene from Dj-BS (S7 and S8 Files) and performed whole-mount *in situ* hybridization. The results revealed that the *DYNLRB-like* gene was primarily expressed in pharynx and intestinal tissues in response to the challenge with LPS and PGN (S9 File), which showed similar expression pattern with the previously study after fed with *L*. *pneumophila* and *S*. *aureus* [6]. This showed that LPS and PGN could simulate bacteria in planarian immune response. The *DYNLRB-like* gene belongs to the km23/DYNLRB/LC7/robl family of dynein light chains. Reports [52] found that km23 plays an important role in TGF-β signal transduction in mammalian cells and zebrafish ovarian follicle cells. In drosophila, the *roblz* deletion mutant shows a female sterile phenotype [53]. The mechanism of the *DYNLRB-like* gene in planarian merits further study.

### Quantitative real-time PCR analysis of immune related gene transcripts in RIG-I-like receptor signaling pathway

**Fig 4:**
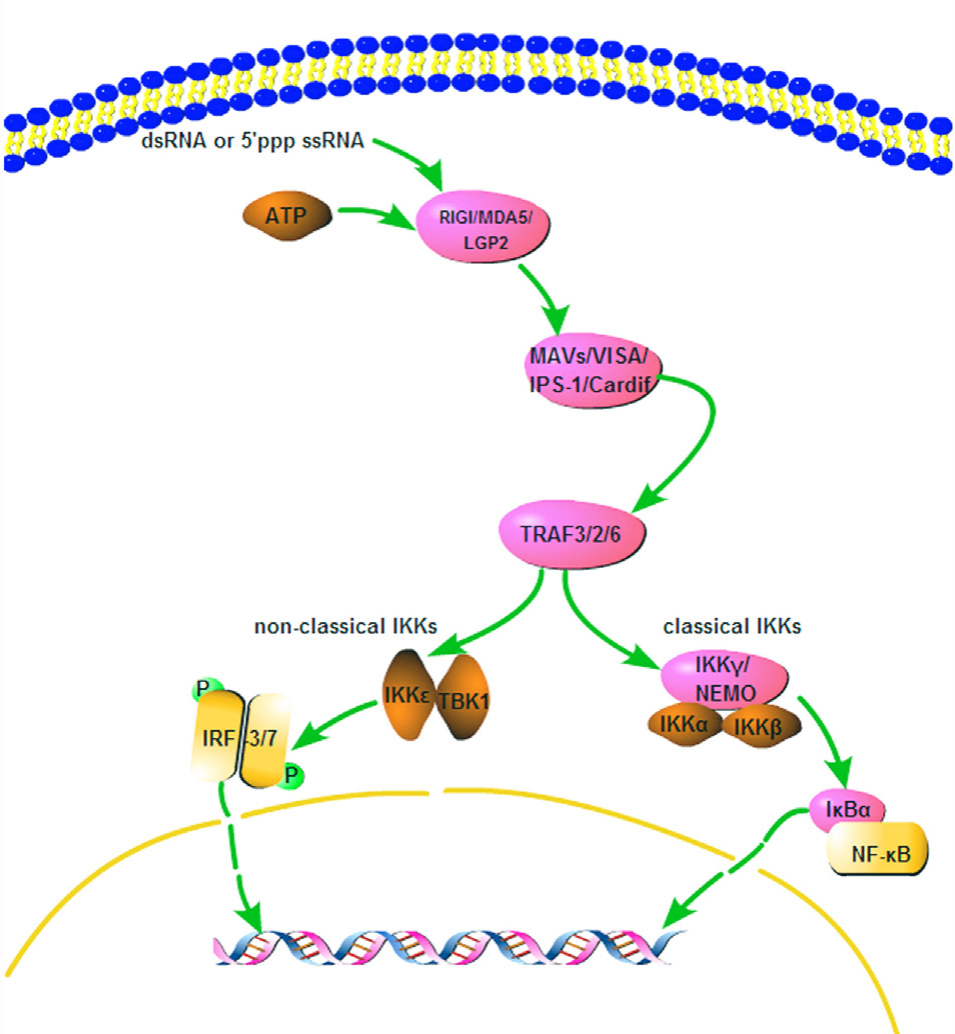
RIG-I mediated signal transduction pathway.

**Fig 5:**
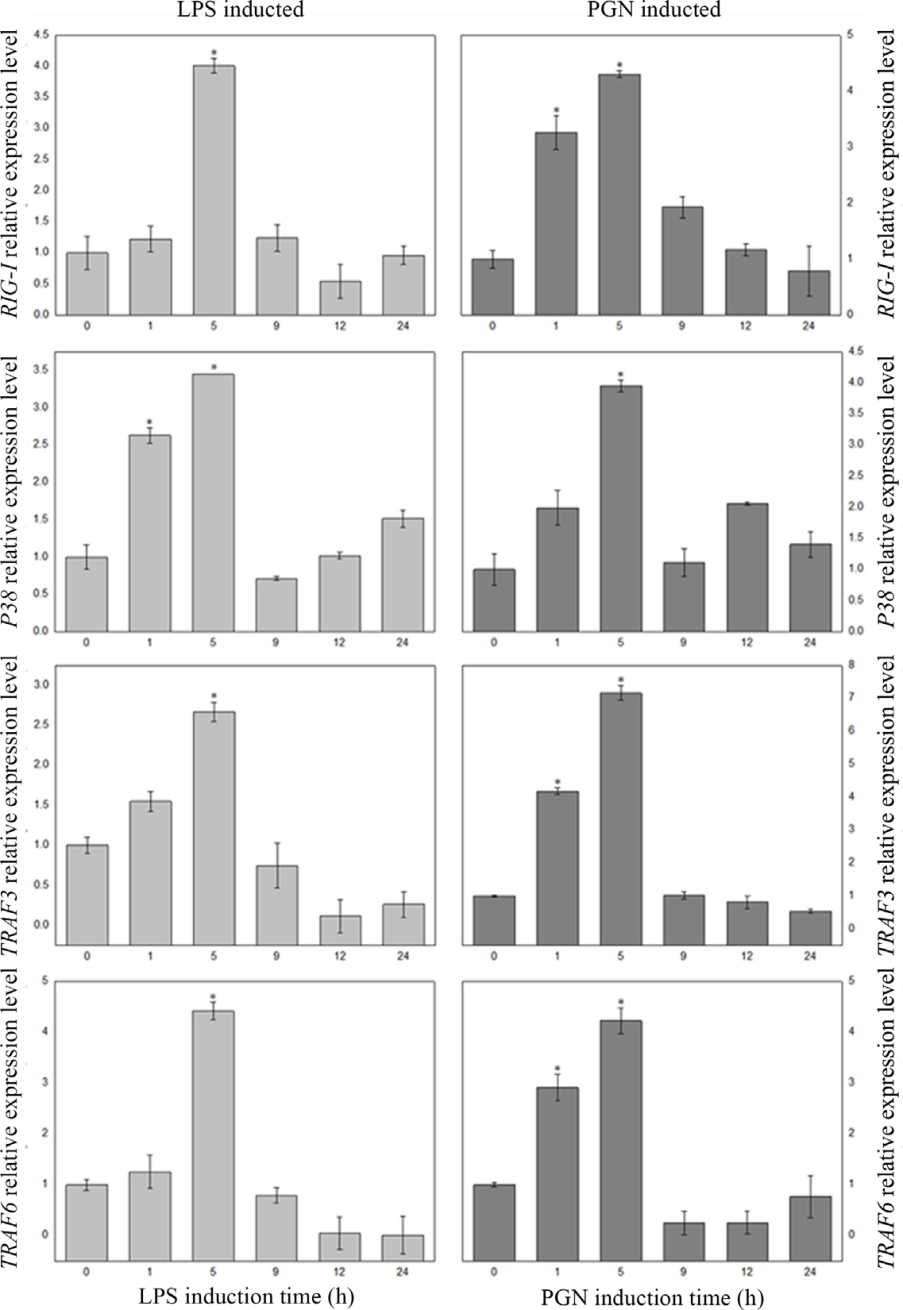
Expression analysis of 4 genes related to immune response in Dj-BS. The mRNA level of each gene was determined by real-time PCR. Values are given as the means ± S.D. (N = 5), and * indicates a significant difference, with P< 0.01.

To investigate whether these four genes (*RIG-I*, *TRAF3*, *TRAF6*, *P38*) related to RIG-I-like receptor signaling pathway **(Fig 4)** are involved in the immune response process of Dj-BS, real-time qRT-PCR was performed (S7 and S8 Files). Animals were inducted with LPS (10 μg/ml) or PGN (10 μg/ml), and gene expression patterns were measured at six different time points. As shown in **Fig 5**, the tendencies of gene expression of all genes stimulated with different PAMPs (LPS and PGN) were similar. Gene expression increased upon simulation and the level of expression peaked (P<0.01) at 5 h, compared with the expression level at 0 h. The expression gradually declined after 9 h, and leveled off after a slight fluctuation. The concentration of stimulus after 5 h of these genes increased three- to five-fold time compared with concentration of homeostasis stage, implying that these genes belong to Group 2 APP [54]. As expected, these candidate genes are acute phase protein whose the levels may increase and decrease markedly within 24 h in response to pathogens infection. Then, in order to maintain homeostasis balance these genes will return to normal levels. The up-regulated gene expression at 5 h, which was similar with the temporal expression patterns of *CgRIG-I* in poly(I:C) infected *C*. *gigas* [55], demonstrates that these genes are involved in the corresponding immune response pathway upon LPS and PGN stimulation. Subsequent results of candidate genes expression changes after bacteria stimulation further evidence that these genes involve in the response to bacterial infection (S10 File). Reports revealed that TRAF6, TRAF3 and P38 play a pivotal role in the RIG-I-like receptor signaling pathway [44,46,47]. Previously study showed that *CgTRAF3* responds to bacterial and viral stimulation [56]. *CgTRAF3-L* was up regulated at 6 h after injection with *V*. *anguillarum*, and *CgTRAF3-S* had a significantly increase at 6 h post-injection with OsHV-1 [53]. In addition, *duTRAF6* knockdown by RNAi observably reduced poly(I:C) and Sendai virus-induced NF-κB activation in DEFs, suggesting that *duTRAF6* functions as a positive regulator in response to nucleic acid challenge [57]. Similar results were observed in *E*. *coioides*, where *EcTRAF6* activates *NF-jB* and has an important role in host defense against parasitic infections [58]. We suspected the activation of *RIG-I* of Dj-BS upon LPS and PGN simulation might activate downstream signaling pathways that lead to up-regulation of the expression of *TRAF3*, *TRAF6* and *P38*. However, some previous studies showed that *RIG-I* associated with the infection of virus, which mainly recognizes the dsRNA of virus [37,42,59–61]. Even though *CgRIG-I* shares significant similarity with Dj-BS RIG-I gene (68%), in PAMPs infected *C*. *gigas*, no prominent increase in expression was observed after LPS and PGN stimulation [55]. The functional amino acids of RIG-I may have mutated during evolution, leading to the loss of binding affinity with bacteria and other pathogens. The mechanism of LPS and PGN participating in RIG-I pathway in Dj-BS merits further study.

### Sequence alignments of candidate RIG-I gene

**Fig 6:**
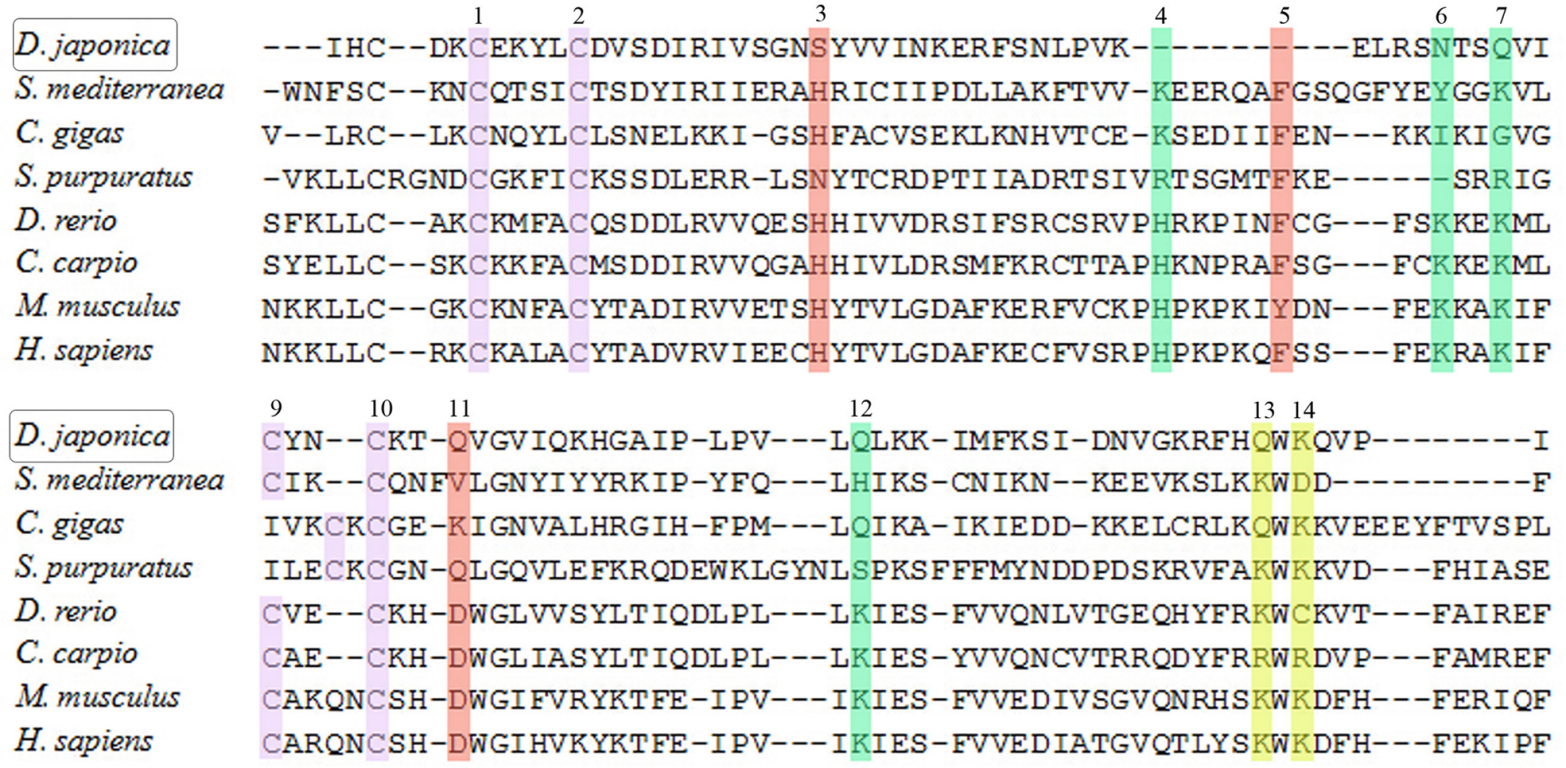
MAFFT amino acid alignment of RIG-I CTD domain between Dj-BS and other species. The amino acids with different functions were marked by blue, green, red and yellow boxes respectively.

Elevated gene expression of *RIG-I* gene was observed, for the first time in this study, upon LPS and PGN stimulation in animals. Previous studies have shown that RIG-I is a cytosolic sensor of viral RNAs and plays crucial roles in immune response [33]. It binds RNAs in its C-terminal domain (CTD) [28,29]. Lu *et al*. found that, in RIG-I-like receptors (RLRs), three clusters of residues participate in RNA binding with different mechanisms [62]. To understand how RIG-I interacts with LPS and PGN, we performed multiple alignment of the CTD domain of RIG-I from Dj-BS with that from *Homo sapiens* and other species using MAFFT. We searched the RIG-I amino acids of different species including *Danio rerio* (KM281808.1), *Cyprinus carpio* (HQ850439.1), *Homo sapiens* (AF038963.1), *Mus musculus* (NM172689.3), *Crassostrea gigas* (AGQ42556.1), *Strongylocentrotu purpuratus* (XP782422.3) from Genbank and *Schmidtea mediterranea* (SMU15039440) from http://smedgd.neuro.utah.edu/. The amino acid sequences of RIG-I in these species are very conservative, and share a high identity of up to 72%; among these, the identity between *H*.*sapiens* and Dj-BS is 70.7%. Our results, as shown in **Fig 6**, revealed that Dj-BS also had these four conservative cysteines (C1, C2, C8, C9) as in *H*. *sapiens*, as described in Zhang *et al*. [55]. In the first cluster of residues, which assists in recognizing the 5'-ppp of dsRNA, all four residues (V4, N6, Q7, Q11) from Dj-BS are different from those in *H*. *sapiens*. Similarly, two residues (S3, Q10) in the third cluster are different and the other residue (F5) is not present in Dj-BS. In the second cluster, one residue (K13) is conservative, and at the position for the other (K12), a Q is present instead. The sequence and conformational change might be the cause of LPS and PGN binding and stimulation. Further experiments, such as binding assays, should be carried out to understand the molecular basis of LPS and PGN stimulation through RIG-I.

## Conclusion

In this study, we performed a *de novo* transcriptome analysis of Dj-BS based on the high throughput sequencing technology. Through function annotation of the assembled transcripts, a number of critical and conserved signaling pathways and genes related to innate immune response were identified. A *DYNLRB-like* gene was cloned, and whole-mount *in situ* hybridization was used to verify the function of the *DYNLRB-like* gene in the immune response. We also demonstrated LPS and PGN could simulate bacteria in planarian immune response. Four candidate genes were further studied, which are involved in the RIG-I-like receptor signaling pathway, including *RIG-I*, *TRAF3*, *TRAF6* and *P38*. The gene expressions of these factors were elevated upon pathogen stimulation, indicating a possible role in immune response. In addition, the annotated transcripts provide a useful resource for subsequent investigation of molecular mechanism of innate immune system in planarians.

## Materials and Methods

### Animals

*D*. *japonica* (clonal strain BS, called Dj-BS) was collected from a fountain in Boshan district, Zibo city, Shandong province, China. The animals were reared in deionized water mixed with autoclaved tap water at a ratio of 1:1 at 20°C. The animals were fed on beef liver twice per week. The animals were starved for 3 to 5 days before the start of the experiments. The Institutional Review Board of Shandong University of Technology approved this study. This study did not involve endangered or protected species. Planarians collection and experiments were approved by the Animal Care and Use Committee at Shandong University of Technology.

### RNA extraction, cDNA library preparation and transcriptome sequencing

Total RNA was extracted with TRIZOL reagent (Invitrogen, California, USA) from adult planarians frozen in liquid nitrogen according to the manufacturer’s protocol. mRNA was extracted through magnetic beads with Oligo(dT) (Dynabeads Oligo(dT)^25^, Invitrogen, California, USA). mRNAs were fragmented into short pieces for synthesis of double-strand cDNAs. cDNA libraries were prepared using a TruSeq RNA sample prep Kit (Illumina, San Diego, CA, USA). cDNAs were purified and quantified using Certified Low Range Ultra Agarose (Bio-Red, Hercules, California, USA) and TBS380 Picogreen (Invitrogen, California, USA). Clusters were generated on acBot (Illumina, San Diego, California, USA) using TruSeq PE Cluster Kit v3-cBot-HS (Illumina, San Diego, California, USA). The flow cell hybridized with sequencing primers was installed onto the IlluminaHiSeq2000, and the sequencing-by-synthesis was carried out using HiSeq2000 TruSeq SBS Kit v3-HS (200 cycles) (Illumina, San Diego, CA, USA).

### *De novo* transcriptome assembly

Firstly, raw image data obtained from IlluminaHiSeq2000 sequencing were converted to sequenced reads in the format of FASTQ through base calling. After initial quality evaluation, these raw reads were pre-processed by stripping the adaptors using SeqPrep (https://github.com/jstjohn/SeqPrep). The remaining sequences with a length of less than 20 bp under the specific parameters (-q = 20, -minlen = 20) were also removed using Sickle (https://github.com/najoshi/sickle). *De novo* assembly of high quality reads was performed using Trinity (http://trinityrnaseq.sourceforge.net/), which is composed of three independent software modules: Inchworm, Chrysalis and Butterfly [39]. By Inchworm, RNA-seq data was assembled into unique sequences through establishing a k-mer graph (K = 25). By Chrysalis, the contigs generated in the previous step were clustered, and a de Bruijn graph was constructed for each cluster. Subsequently, by Butterfly, these Bruijn graphs were processed in parallel and the paths were traced according to reads and pairs of reads within graphs to obtain full-length transcripts for alternative splicing isoforms and distinguish transcripts of paralogous genes. Finally, the distributed situation of length of result sequences was counted and analyzed.

### Functional annotation and Gene Ontology and KEGG pathway annotation

ORF prediction of assembled transcripts was carried out using Trinity (http://trinityrnaseq.sourceforge.net/analysis/extract_proteins_from_trinity_transcripts.html) before functional annotation. Predicted protein sequences with ORFs and nucleic acid sequences without ORFs were aligned with Genbank NR, String and Gene databases using BLAST (Version 2.2.25 with an E-value ≤ 1e-5). Top-Hit sequences were retrieved from NT, NR and String and were annotated according to the Gene Ontology (GO) databases (http://www.geneontology.org/) using the Blast2GO program (http://www.blast2go.com/b2ghome) [42]. All assembled transcripts were aligned with GENES (http://www.genome.jp/kegg/genes.html) using BLAST algorithm (Blastx/Blastp 2.2.24+) based on KEGG databases (http://www.genome.jp/kegg/), and the specific biological pathways shared by multiple genes were assigned accordingly.

### Whole-mount *in situ* hybridization (WISH)

Whole-mount *in situ* hybridization was performed as previously described [63]. Planarians samples were observed with a Nikon SMZ 1500 stereomicroscope (Nikon, Japan). Planarians were exposed and challenged with 10 μg/ml LPS and 10 μg/ml PGN in sterile water. After 6 h of inducement, the animals were washed and used for experiment.

### Real-Time Quantitative Reverse Transcription PCR (real-time qRT-PCR) analysis

Total RNA was extracted with TRIZOL reagent from adult planarians stimulated by LPS (10 μg/ml), PGN (10 μg/ml), Gram-negative bacteria (*Escherichia coli* 44102 and *Pseudomonas aeruginosa* 10104, 10^7^/ml) and Gram-positive bacteria (*Staphylococcus aureus* 26003 and *Bacillus subtilis* 63501, 10^7^/ml), respectively, at different time points (0, 1, 5, 9, 12 and 24 h). RNA was reverse transcribed into cDNA, which was used as a template in the PCR according to the protocols of Revert Aid First Strand cDNA Synthesis Kit (Thermo Scientific, Waltham, Massachusetts, USA). PCR amplification was performed on an ABI 7500 real-time PCR system using a Fast Start Universal SYBR Green Master (Rox) (Roche, Basel, Switzerland). The primers for PCR were designed by Primer 5.0. Values are given as the means ± S.D. (N = 5), and * indicates a significant difference, with P< 0.01.

## Supporting Information

S1 File Quality control chart of raw reads. (DOCX)

S2 File Sequence length distribution of assembled results. (DOCX)

S3 File The results of GO analysis on immune related genes. (TXT)

S4 File The annotation of planarian Dj-BS transcriptome. (XLS)

S5 File Comparison of GO annotation between Dj-BS and Dj-Qin transcriptome. (XLSX)

S6 File The results of KEGG analysis on immune related genes and pathways. (ZIP)

S7 File Sequence information of four genes within the assembled results. (TXT)

S8 File Primers of immune related genes selected for detection by qRT-PCR. (TXT)

S9 File *In situ* hybridization and sequence alignment results of *DYNLRB-like* gene. (TIF)

S10 File Expression analysis of 4 genes after bacteria stimulation in Dj-BS. (ZIP)

## References

[1] Alevarado AS, Newmark NP. Double-stranded RNA specifically disrupts gene expression during planarian regeneration. Proc Natl Acad Sci USA. 1999; 96(9): 5049–5054. 10220416 10.1073/pnas.96.9.5049 PMC21814

[2] Baguna J, Salo E, Collet J, Auladell MC, Ribas M. Cellular, molecular and genetic approaches to regeneration and pattern formation in planarians. Fortschr Zool. 1988; 36: 65–78.

[3] Salo E. The power of regeneration and the stem cells kingdom: The fresh water planarian *Schmidtea mediterranea* (Platyhelminth). Bioessays. 2006; 28(5): 546–559. 16615086 10.1002/bies.20416

[4] Handberg-Thorsager M, Fernandez E, Salo E. Stem cells and regeneration in planarians. Front Biosci. 2008; 13: 6374–6394. 18508666 10.2741/3160

[5] Forsthoefel DJ, Newmark PA. Emerging patterns in planarian regeneration. Curr Opin Genet Dev. 2009; 19(4): 412–420. 10.1016/j.gde.2009.05.003 19574035 PMC2882238

[6] Abnave P, Mottola G, Gimenez G, Boucherit N, Trouplin V, Torre C, et al Screening in planarians identifies MORN2 as a key component in LC3-associated phagocytosis and resistance to bacterial infection. Cell Host Microbe. 2014; 16(3): 338–350. 10.1016/j.chom.2014.08.002 25211076

[7] Petersen CP. Planarian Resistance to Blades and Bugs. Cell Host Microbe. 2014; 16: 271–272. 10.1016/j.chom.2014.08.016 25211069

[8] Bachere E, Gueguen Y, Gonzalez M, de Lorgeril J, Garnier J, Romestand B. Insights into the anti-microbial defense of marine invertebrates: the penaeid shrimps and the oyster *Crassostrea gigas*. Immunol Rev. 2004; 198: 149–168. 15199961 10.1111/j.0105-2896.2004.00115.x

[9] Mialhe E, Bachere E, Boulo V, Cadoret JP, Rousseau C, Cedeno V. Future of biotechnology-based control of disease in marine invertebrates. Mol Mar Biol Biotechnol. 1995; 4(4): 275–283. 8541979

[10] Tseng IT, Chen JC. The immune response of white shrimp *Litopenaeus vannamei* and its susceptibility to *Vibrio alginolyticus* under nitrite stress. Fish Shellfish Immunol. 2004; 17(4): 325–333. 15312659 10.1016/j.fsi.2004.04.010

[11] Medzhitov R, Janeway CA Jr. Innate immunity: the virtues of a nonclonal system of recognition. Cell. 1997; 91(3): 295–298. 9363937 10.1016/s0092-8674(00)80412-2

[12] Medzhitov R, Janeway CA Jr. Innate immunity: impact on the adaptive immune response. Curr Opin Immunol. 1997; 9(1): 4–9. 9039775 10.1016/s0952-7915(97)80152-5

[13] Hoffmann JA, Kafatos FC, Janeway CA, Ezekowitz RA. Phylogenetic perspectives in innate immunity. Science. 1999; 284(5418): 1313–1318. 10334979 10.1126/science.284.5418.1313

[14] Lemaitre B, Hoffmann J. The host defense of *Drosophila melanogaster*. Annu Rev Immunol. 2007; 25: 697–743. 17201680 10.1146/annurev.immunol.25.022106.141615

[15] Christophides GK, Vlachou D, Kafatos FC. Comparative and functional genomics of the innate immune system in the malaria vector *Anopheles gambiae*. Immunol Rev. 2004; 198: 127–148. 15199960 10.1111/j.0105-2896.2004.0127.x

[16] Christophides GK, Zdobnov E, Barillas-Mury C, Birney E, Blandin S, Blass C, et al Immunity-related genes and gene families in *Anopheles gambiae*. Science. 2002; 298(5591): 159–165. 12364793 10.1126/science.1077136

[17] Hultmark D. Drosophila immunity: paths and patterns. Curr Opin Immunol. 2003; 15(1): 12–19. 12495727 10.1016/s0952-7915(02)00005-5

[18] Johansson MW. Cell adhesion molecules in invertebrate immunit. Dev Comp Immunol. 1999; 23(4–5): 303–315. 10426424 10.1016/s0145-305x(99)00013-0

[19] Baeg GH, Zhou R, Perrimon N. Genome-wide RNAi analysis of JAK/STAT signaling components in Drosophila. Gene Dev. 2005; 19(16): 1861–1870. 16055650 10.1101/gad.1320705 PMC1186186

[20] Ferrandon D, Imler JL, Hetru C, Hoffmann JA. The Drosophila systemic immune response: sensing and signaling during bacterial and fungal infections. Nat Rev Immunol. 2007; 7(11): 862–874. 17948019 10.1038/nri2194

[21] Leclerc V, Reichhart JM. The immune response of *Drosophila melanogaster*. Immunol Rev. 2004; 198: 59–71. 15199954 10.1111/j.0105-2896.2004.0130.x

[22] Kwon ES, Narasimhan SD, Yen K, Tissenbaum HA. A new DAF-16 isoform regulates longevity. Nature. 2010; 466(7305): 498–502. 10.1038/nature09184 20613724 PMC3109862

[23] Young JA, Dillin A. Mapping innate immunity. Proc Natl Acad Sci USA. 2004; 101(35): 12781–12782. 15328410 10.1073/pnas.0404890101 PMC516471

[24] Morita K, Flemming AJ, Sugihara Y, Mochii M, Suzuki Y, Yoshida S, et al A *Caenorhabditis elegans* TGF-beta, DBL-1, controls the expression of LON-1, a PR-related protein,that regulates polyploidization and body length. EMBO J. 2002; 21(5): 1063–1073. 11867534 10.1093/emboj/21.5.1063 PMC125886

[25] Pujol N, Link EM, Liu LX, Kurz CL, Alloing G, Tan MW, et al A reverse genetic analysis of components of the Toll signaling pathway in *Caenorhabditis elegans*. Curr Biol. 2001; 11(11): 809–821. 11516642 10.1016/s0960-9822(01)00241-x

[26] Lan JF, Zhou J, Zhang XW, Wang ZH, Zhao XF, Ren Q, et al Characterization of an immune deficiency homolog (IMD) in shrimp (*Fenneropenaeus chinensis*) and crayfish (*Procambams clarkii*). Dev Comp Immunol. 2013; 41(4): 608–617. 10.1016/j.dci.2013.07.004 23850721

[27] Li F, Xiang J. Signaling pathways regulating innate immune responses in shrimp. Fish Shellfish Immunol. 2013; 34(4): 973–980. 10.1016/j.fsi.2012.08.023 22967763

[28] Cui S, Eisenacher K, Kirchhofer A, Brzozka K, Lammens A, Lammens K, et al The C-terminal regulatory domain is the RNA 5'-triphosphate sensor of RIG-I. Mol Cell. 2008; 29(2): 169–179. 10.1016/j.molcel.2007.10.032 18243112

[29] Yoneyama M, Fujita T. RNA recognition and signal transduction by RIG-I-like receptors. Immunol Rev. 2009; 227(1): 54–65. 10.1111/j.1600-065X.2008.00727.x 19120475

[30] Xu LG, Wang YY, Han KJ, Li LY, Zhai ZH, Shu HB, et al VISA is an adapter protein required for virus-triggered IFN-beta signaling. Mol Cell. 2005; 19(6): 727–740. 16153868 10.1016/j.molcel.2005.08.014

[31] Kawai T, Takahashi K, Sato S, Coban C, Kumar H, Kato H, et al IPS-1, an adaptor triggering RIG-I- and Mda5-mediated type I interferon induction. Nat Immunol. 2005; 6(10): 981–988. 16127453 10.1038/ni1243

[32] Meylan E, Curran J, Hofmann K, Moradpour D, Binder M, Bartenschlager R, et al Cardif is an adaptor protein in the RIG-I antiviral pathway and is targeted by hepatitis C virus. Nature. 2005; 437: 1167–1172. 16177806 10.1038/nature04193

[33] Seth RB, Sun L, Ea CK, Chen ZJ. Identification and characterization of MAVS, a mitochondrial antiviral signaling protein that activates NF-κB and IRF 3. Cell. 2005; 122(5): 669–682. 16125763 10.1016/j.cell.2005.08.012

[34] Saha SK, Pietras EM, He JQ, Kang JR, Liu SY, Oqanesyan G, et al Regulation of antiviral responses by a direct and specific interaction between TRAF3 and Cardif. EMBO J. 2006; 25(14): 3257–3263. 16858409 10.1038/sj.emboj.7601220 PMC1523175

[35] Bradley JR, Pober JS. Tumor necrosis factor receptor-associated factors (TRAFs). Oncogene. 2001; 20(44): 6482–6491. 11607847 10.1038/sj.onc.1204788

[36] Mikkelsen SS, Jensen SB, Chiliveru S, Melchjorsen J, Julkunen I, Gaestel M, et al RIG-I-mediated activation of p38 MAPK is essential for viral induction of interferon and activation of dendritic cells. J Biol Chem. 2009; 284(16): 10774–10782. 10.1074/jbc.M807272200 19224920 PMC2667765

[37] Yoshida R, Takaesu G, Yoshida H, Okamoto F, Yoshioka T, Choi Y, et al TRAF6 and MEKK1 play a pivotal role in the RIG-I-like helicase antiviral pathway. J Biol Chem. 2008; 283(52): 36211–36220. 10.1074/jbc.M806576200 18984593 PMC2662295

[38] Su ZQ, Ning BT, Fang H, Hong HX, Perkins R, Tong W, et al Next-generation sequencing and its applications in molecular diagnostics. Expert Rev Mol Diagn. 2011; 11(3): 333–343. 10.1586/erm.11.3 21463242

[39] Grabherr MG, Haas BJ, Yassour M, Levin JZ, Thompson DA, Amit I, et al Full-length transcriptome assembly from RNA-Seq data without a reference genome. Nat Biotechnol. 2011; 29(7): 644–652. 10.1038/nbt.1883 21572440 PMC3571712

[40] Qin YF, Fang HM, Tian QN, Bao ZX, Lu P, Zhao JM, et al Transcriptome profiling and digital gene expression by deep-sequencing in normal/regenerative tissues of planarian *Dugesia japonica*. Genomics. 2011; 97(6): 364–371. 10.1016/j.ygeno.2011.02.002 21333733

[41] Altschul SF, Madden TL, Schaffer AA, Zhang J, Zhang Z, Miller W, et al Gapped BLAST and PSI-BLAST: a new generation of protein database search programs. Nucleic Acids Res. 1997; 25(17): 3389–3402. 9254694 10.1093/nar/25.17.3389 PMC146917

[42] Conesa A, Gotz S, Garcia-Gomez JM, Terol J, Talon M, Robles M. Blast2GO: a universal tool for annotation, visualization and analysis in functional genomics research. Bioinformatics. 2005; 21(18): 3674–3676. 16081474 10.1093/bioinformatics/bti610

[43] Kanehisa M, Goto S, Kawashima S, Okuno Y, Hattori M. The KEGG resource for deciphering the genome. Nucleic Acids Res. 2004; 32: D277–280. 14681412 10.1093/nar/gkh063 PMC308797

[44] Peiris TH, Hoyer KK, Oviedo NJ. Innate immune system and tissue regeneration in planarians: An area ripe for exploration. Semin Immunol. 2014; 26(4): 295–302. 10.1016/j.smim.2014.06.005 25082737 PMC4171206

[45] Wang H, Song LS, Li CH, Zhao JM, Zhang H, Ni DJ, et al Cloning and characterization of a novel C-type lectin from Zhikong scallop *Chlamys farreri*. Mol Immunol. 2007; 44(5): 722–731. 16777225 10.1016/j.molimm.2006.04.015

[46] Luo T, Yang HJ, Li F, Zhang XB, Xu X. Purification, characterization and cDNA cloning of a novel lipopolysaccharide-binding lectin from the shrimp *Penaeus monodon*. Dev Comp Immunol. 2006; 30(7): 607–617. 16364436 10.1016/j.dci.2005.10.004

[47] Aspan A, Huanq TS, Cerenius L, Soderhall K. cDNA cloning of prophenoloxidase from the freshwater crayfish *Pacifastacus leniusculus* and its activation. Proc Natl Acid Sci USA. 1995; 92(4): 939–943. 10.1073/pnas.92.4.939 PMC42612 7862669

[48] Soderhall K, Cerenius L, Johansson MW. The prophenoloxidase activating system and its role in invertebrate defence. Ann N Y Acad Sci. 1994; 712: 155–161. 8192329 10.1111/j.1749-6632.1994.tb33570.x

[49] Pang QX, Liu XM, Zhao BS, Jiang YS, Su F, Zhang XF, et al Detection and characterization of phenoloxidase in the freshwater planarian *Dugesia japonica*. Comp Biochem Physiol B. 2010; 157(1): 54–58. 10.1016/j.cbpb.2010.05.002 20462518

[50] Liang YJ, Pan AX, Zhang SC, Zhang Y, Liu MY. Cloning, distribution and primary immune characteristics of amphioxus alpha-2 macroglobulin. Fish Shellfish Immunol. 2011; 31(6): 963–969. 10.1016/j.fsi.2011.08.014 21903171

[51] Yang JL, Wang LL, Zhang H, Qiu LM, Wang H, Song L. C-type lectin in *Chlamys farreri* (CfLec-1) mediating immune recognition and opsonization. PLOS ONE. 2011; 6(2): e1089. 10.1371/journal.pone.0017089 PMC3039652 21347232

[52] Jin QY, Gao GF, Mulder KM. Requirement of a dynein light chain in transforming growth factor β signaling in zebrafish ovarian follicle cells. Mol Cell Endocrinol. 2012; 348(1): 233–240. 10.1016/j.mce.2011.08.029 21920407 PMC3205241

[53] Bowman A, Patel-King R, Benashski S, McCaffery J, Goldstein L, King SM. Drosophila roadblock and Chlamydomonas LC7: a conserved family of dynein-associated proteins involved in axonal transport, flagellar motility, and mitosis. J Cell Biol. 1999; 146(1): 165–180. 10402468 PMC2199740

[54] Bayne CJ, Gerwick L, Fujiki K, Nakao M, Yano T. Immune-relevant (including acute phase) genes identified in the livers of rainbow trout, Oncorhynchus mykiss, by means of suppression subtractive hybridization. Dev Comp Immunol. 2001; 25: 215–217. 10.1016/s0145-305x(00)00057-4 11164886

[55] Zhang Y, Yu ZN, Li J, Tong Y, Zhang YH, Yu ZN. The first invertebrate RIG-I-like receptor (RLR) homolog gene in the pacific oyster *Crassostrea giga*s. Fish Shellfish Immunol. 2014; 40(2): 466–471. 10.1016/j.fsi.2014.07.029 25107697

[56] Huang BY, Zhang LL, Du YS, Li L, Qu T, Meng J, Alternative splicing and immune response of *Crassostrea gigas* tumor necrosis factor receptor-associated factor 3. Mol Biol Rep. 2014; 41(10): 6481–6491. 10.1007/s11033-014-3531-9 25012913

[57] Zhai YJ, Luo F, Chen YS, Zhou SS, Li ZL, Liu M, et al Molecular characterization and functional analysis of duck TRAF6. Dev Comp Immunol. 2015; 49(1): 1–6. 10.1016/j.dci.2014.11.006 25445905

[58] Li YW, Li X, Xiao XX, Zhao F, Luo XC, Dan XM, et al Molecular characterization and functional analysis of TRAF6 in orange-spotted grouper (*Epinephelus coioides*). Dev Comp Immunol. 2014; 44(1): 217–225. 10.1016/j.dci.2013.12.011 24378225

[59] Feng H, Liu H, Kong RQ, Wang L, Wang YP, Hu W, et al Expression profiles of carp IRF-3/-7 correlate with the up-regulation of RIG-I/MAVS/TRAF3/TBK1, four pivotal molecules in RIG-I signaling pathway. Fish Shellfish Immunol. 2011; 30(4–5): 1159–1169. 10.1016/j.fsi.2011.03.002 21385615

[60] Nie L, Zhang Y, Dong WR, Xiang LX, Shao JZ. Involvement of zebrafish RIG-I in NF-κB and IFN signaling pathways: insights into functional conservation of RIG-I in antiviral innate immunity. Dev Comp Immunol. 2015; 48(1): 95–101. 10.1016/j.dci.2014.09.008 25265425

[61] Mao M, Yu M, Tong JH, Ye J, Zhu J, Huang QH, et al RIG-E, a human homolog of the murine Ly-6 family, is induced by retinoic acid during the differentiation of acute promyelocytic leukemia cell. Proc Natl Acad Sci USA. 1996, 93(12): 5910–5914. 8650192 10.1073/pnas.93.12.5910 PMC39161

[62] Lu C, Xu H, Ranjith-Kumar CT, Brooks MT, Hou TY, Hu FQ, et al The structural basis of 5' triphosphate double-stranded RNA recognition by RIG-I C-terminal domain. Structure. 2010; 18(8): 1032–1043. 10.1016/j.str.2010.05.007 20637642 PMC2919622

[63] Zeng A, Li YQ, Wang C, Han XS, Li G, Wang JY, et al Heterochromatin protein 1 promotes self-renewal and triggers regenerative proliferation in adult stem cells. J Cell Biol. 2013; 201(3): 409–423. 10.1083/jcb.201207172 23629965 PMC3639387

