# Supplementary material for: De Novo Transcriptome Analysis Provides Insights into Immune Related Genes and the RIG-I-Like Receptor Signaling Pathway in the Freshwater Planarian (Dugesia japonica)
Source: PLoS One. 2016 Mar 17;11(3):e0151597. doi: 10.1371/journal.pone.0151597 (PMC4795655; doi:10.1371/journal.pone.0151597)
Supplement: S1 File — (DOCX) [file pone.0151597.s001.docx]

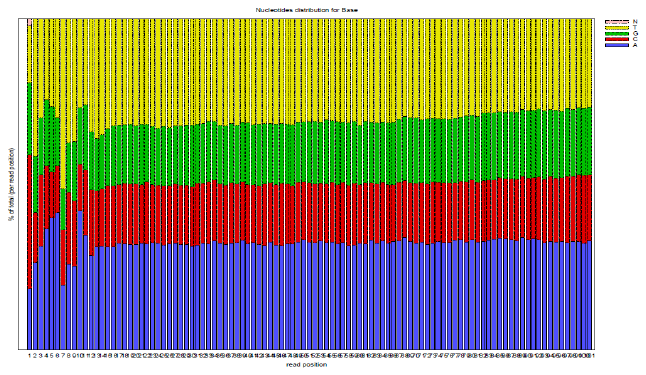


**Distribution diagram of nucleic acid of raw reads (also known as GC deviation chart).** Abscissa represents the nucleic acid of each reads, ordinate is the percentage of A, T, C and G within each reads.


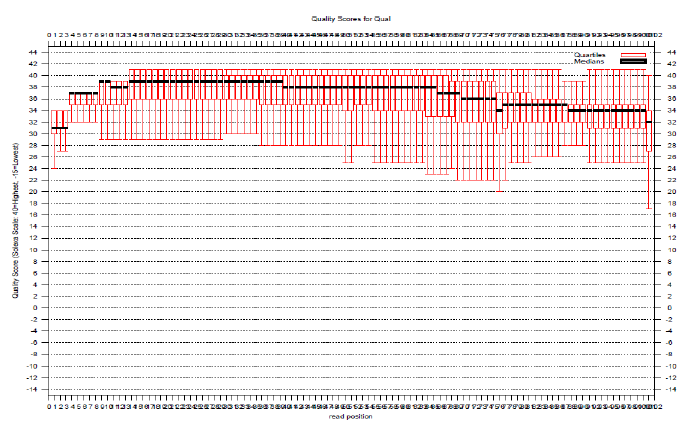


**Distribution diagram of nucleic acid quality of raw reads.** Abscissa represents the nucleic acid of each reads, ordinate reveals the quality of nucleic acid in each reads (Solexa Scale: 40=Highest, -15=Lowest). The range specified with vertical red line “I” represent comprehensive quality of all reads bases, and overstriking line is the median of quality value.
