# Supplementary material for: De Novo Transcriptome Analysis Provides Insights into Immune Related Genes and the RIG-I-Like Receptor Signaling Pathway in the Freshwater Planarian (Dugesia japonica)
Source: PLoS One. 2016 Mar 17;11(3):e0151597. doi: 10.1371/journal.pone.0151597 (PMC4795655; doi:10.1371/journal.pone.0151597)
Supplement: S2 File — (DOCX) [file pone.0151597.s002.docx]

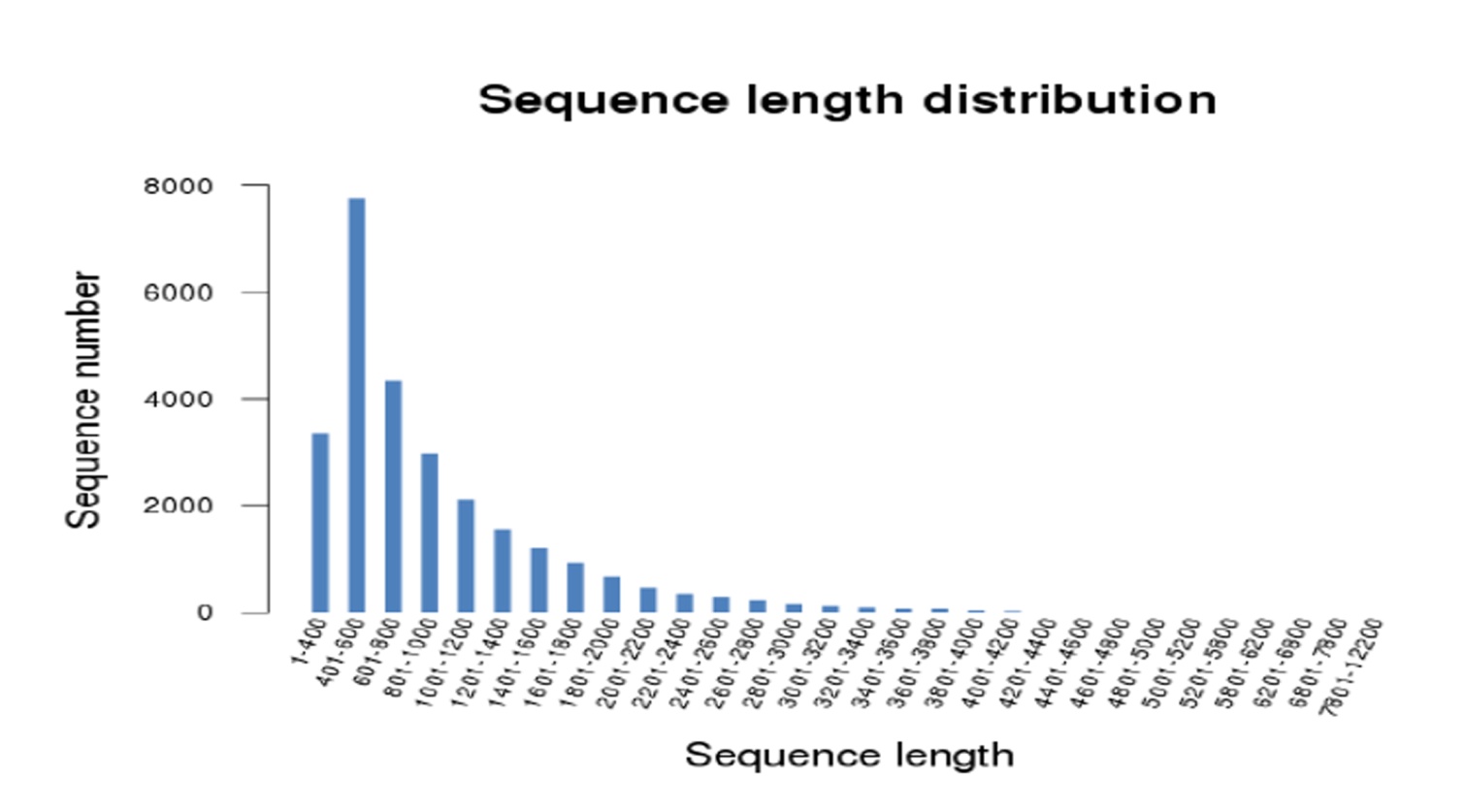


Table 1B Sequence length distribution of assembled results

| Length of isogene Number ofisogenePercentage |
| --- |
| 1-400 3372 12.41％  401-600 7767 28.58％  601-800 4352 16.01％  801-1000 2983 10.97％  1001-1200 2126 7.82％  ……  ALL 27180 100％ |
