# Supplementary figures and images for: De Novo Transcriptome Analysis Provides Insights into Immune Related Genes and the RIG-I-Like Receptor Signaling Pathway in the Freshwater Planarian (Dugesia japonica)
Source: PLoS One. 2016 Mar 17;11(3):e0151597. doi: 10.1371/journal.pone.0151597 (PMC4795655; doi:10.1371/journal.pone.0151597)

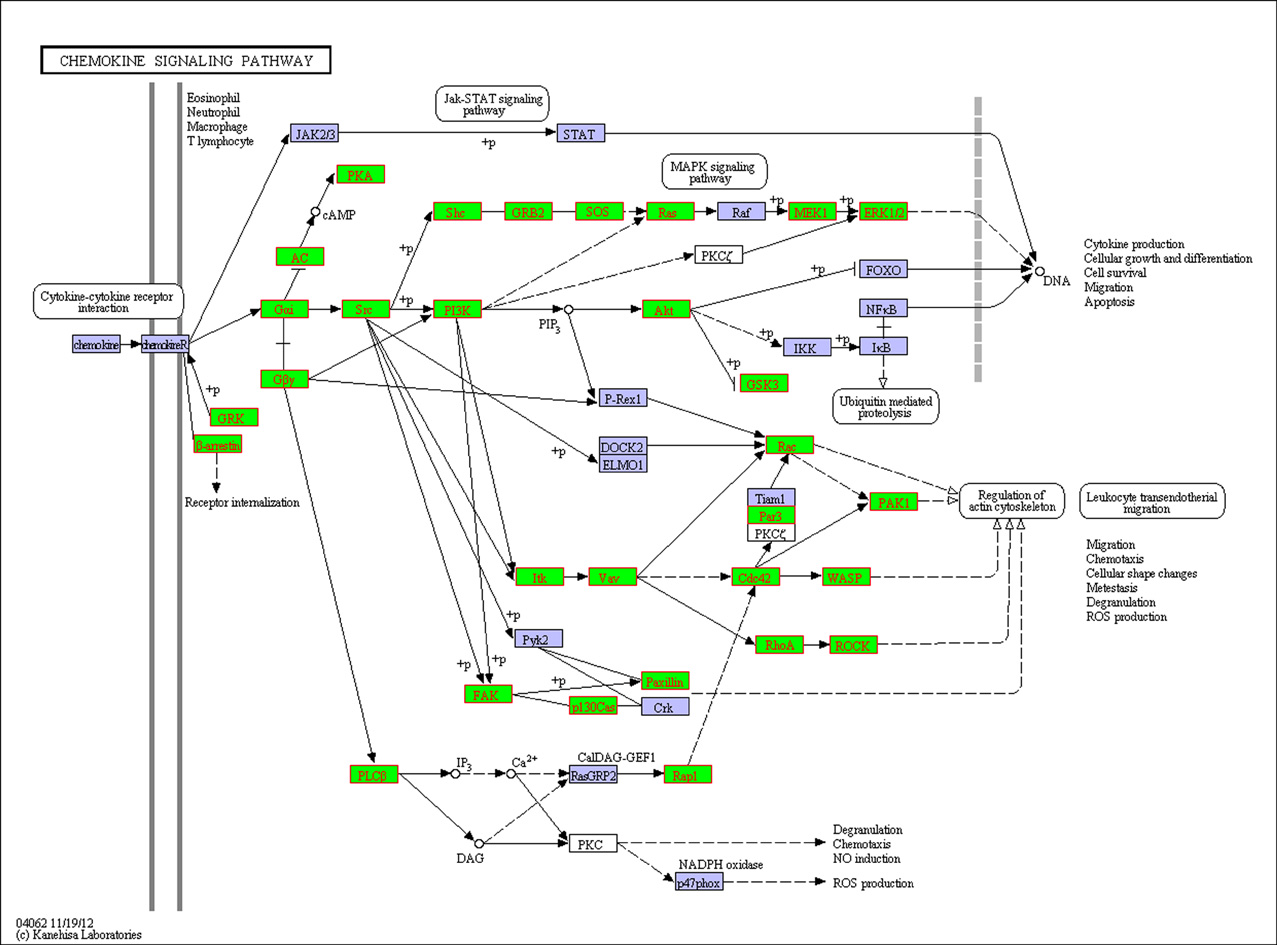

Supplement: S6 File — (ZIP) [file pone.0151597.s006.zip › S file 6/S4-1.jpg]

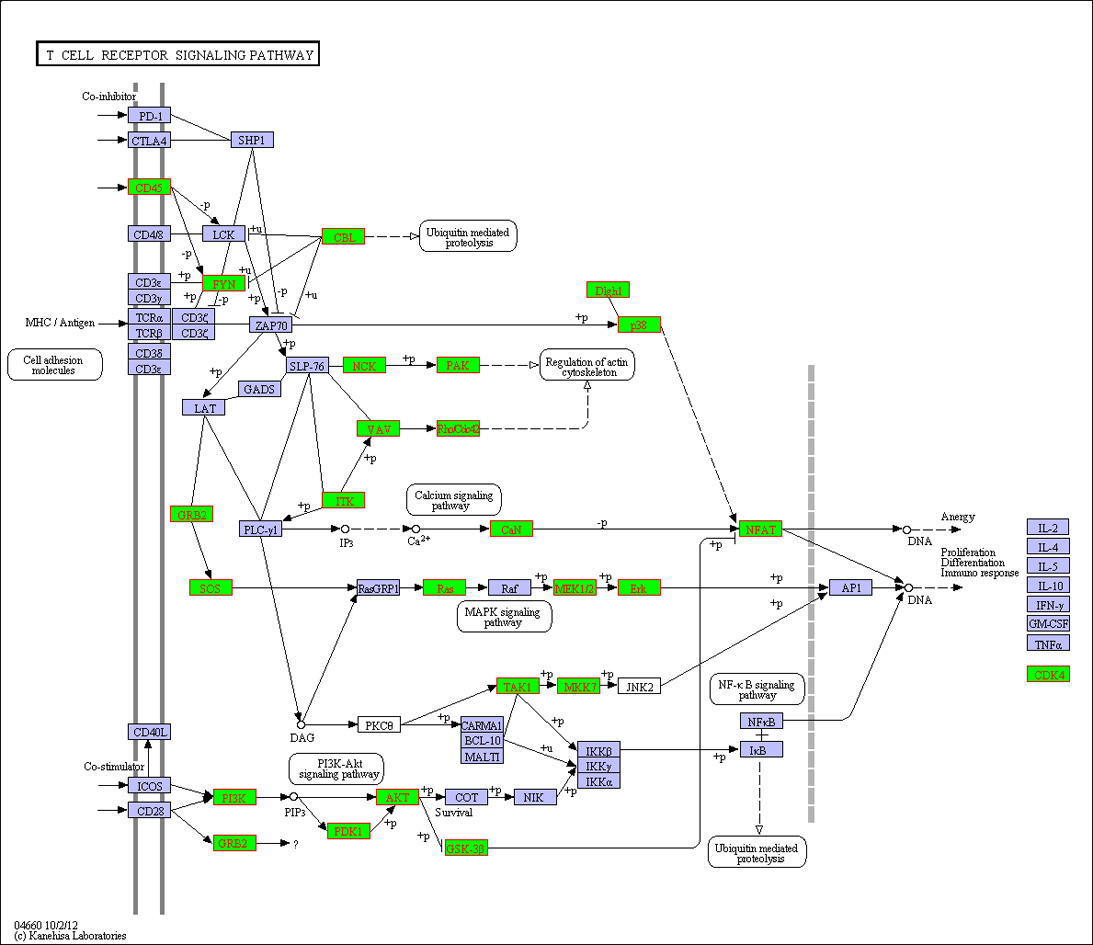

Supplement: S6 File — (ZIP) [file pone.0151597.s006.zip › S file 6/S4-10.jpg]

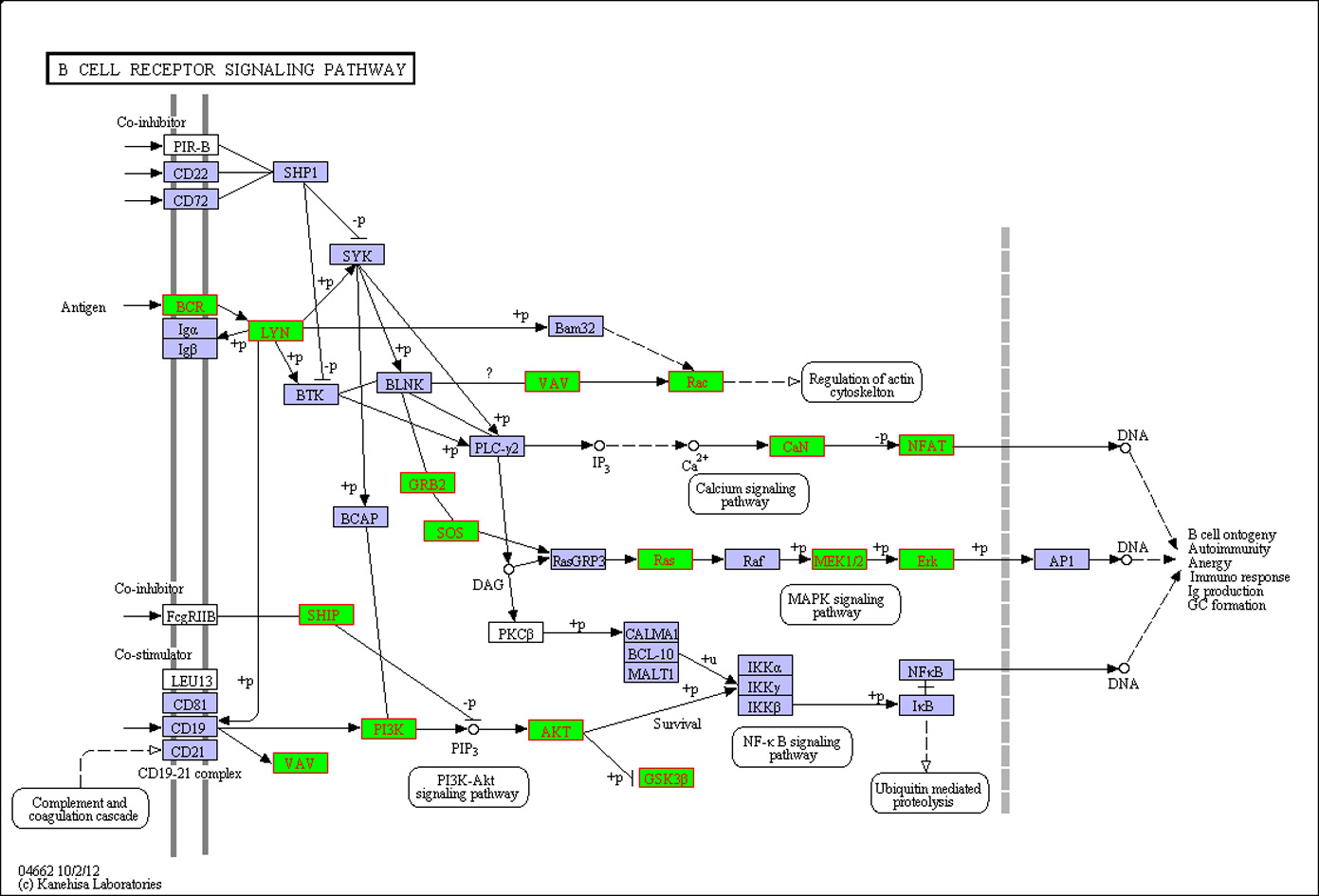

Supplement: S6 File — (ZIP) [file pone.0151597.s006.zip › S file 6/S4-11.jpg]

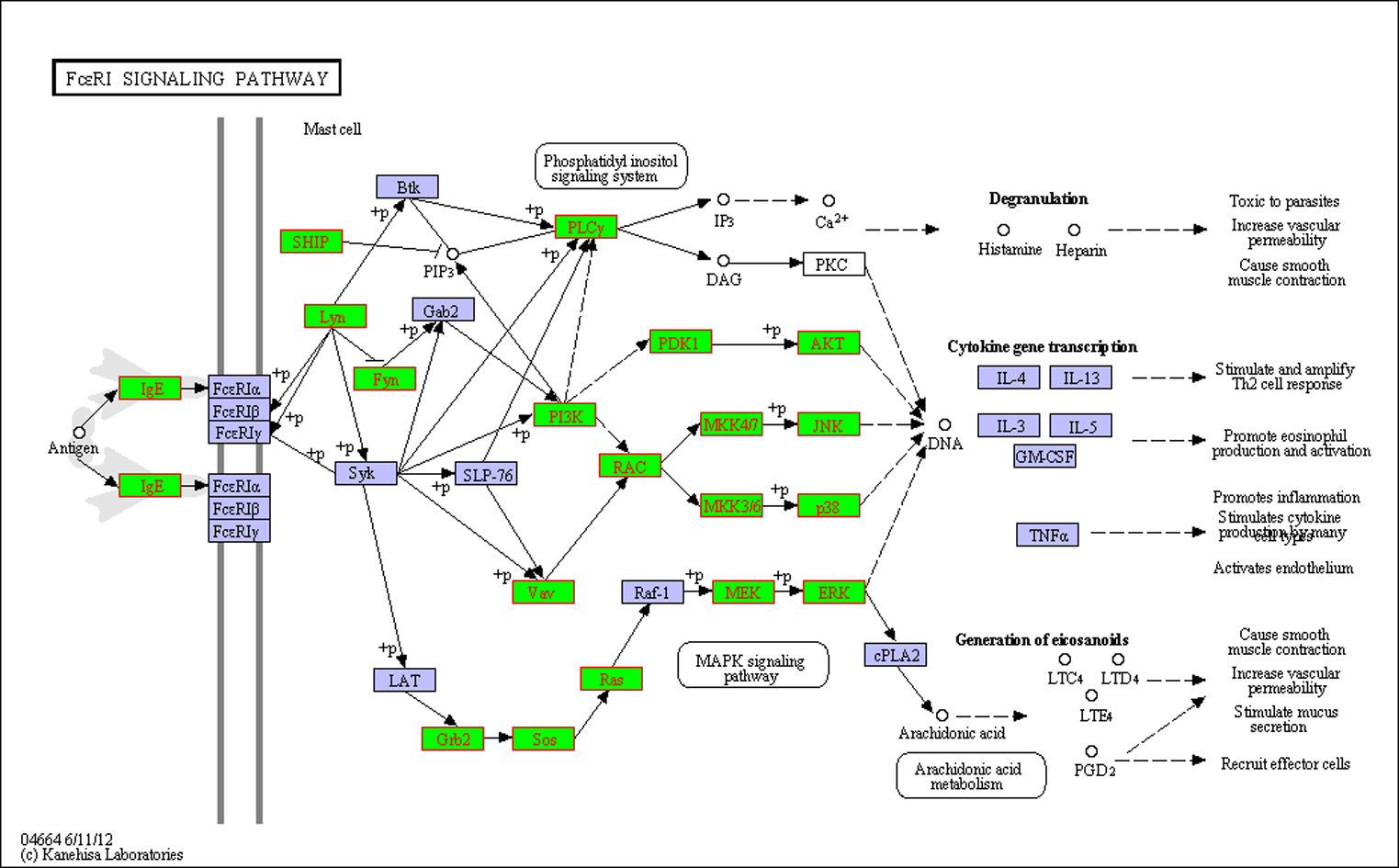

Supplement: S6 File — (ZIP) [file pone.0151597.s006.zip › S file 6/S4-12.jpg]

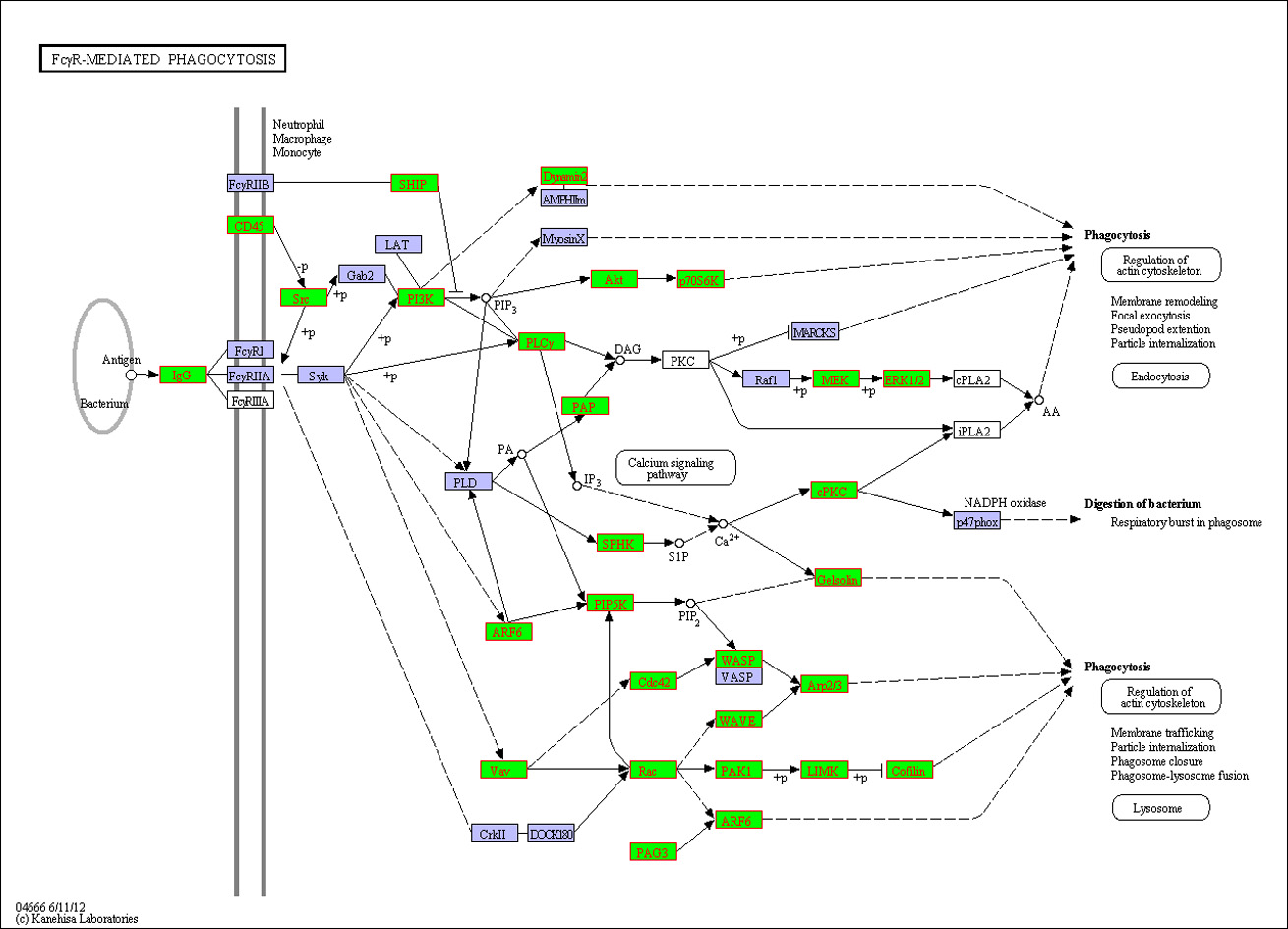

Supplement: S6 File — (ZIP) [file pone.0151597.s006.zip › S file 6/S4-13.jpg]

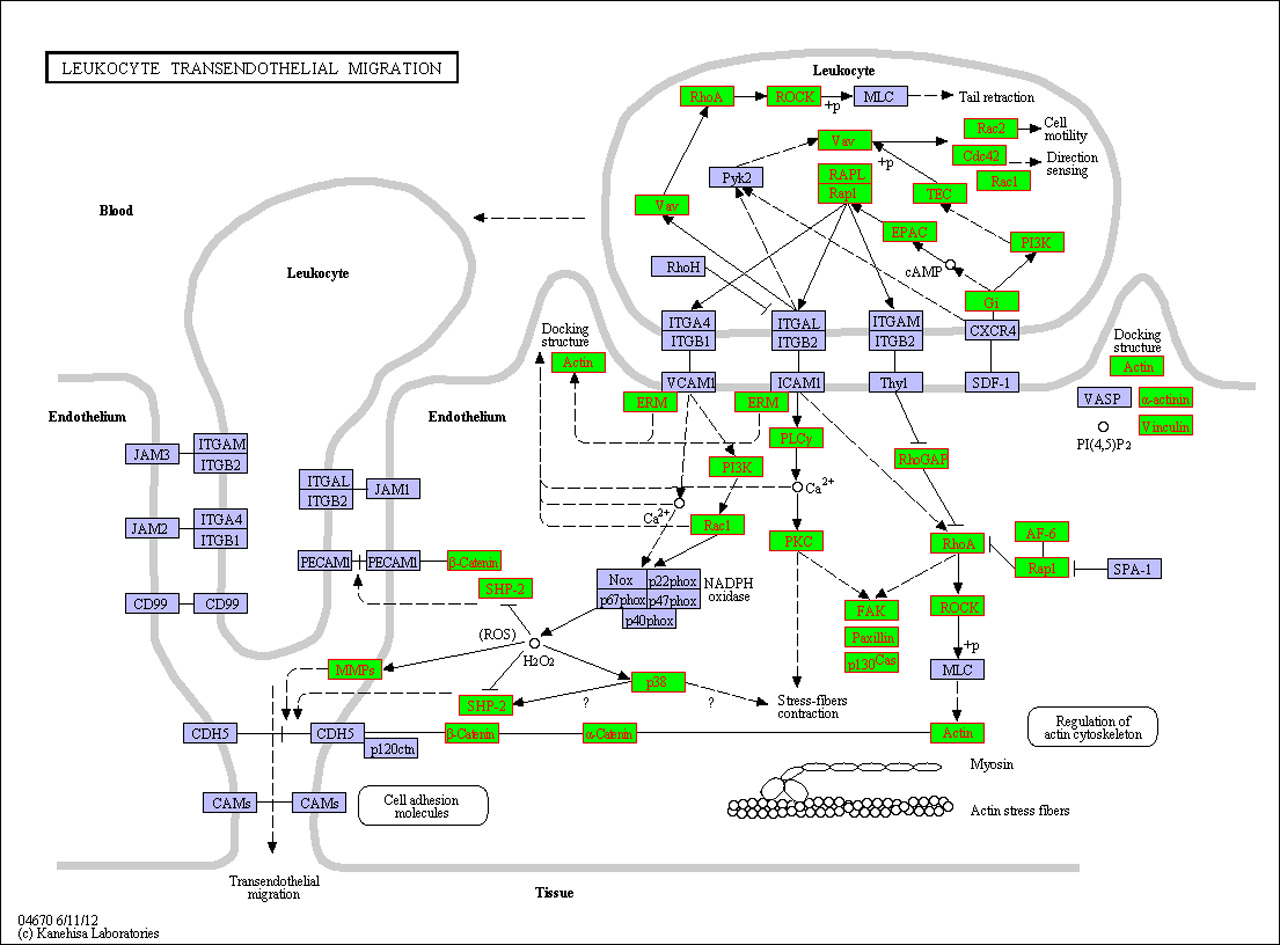

Supplement: S6 File — (ZIP) [file pone.0151597.s006.zip › S file 6/S4-14.jpg]

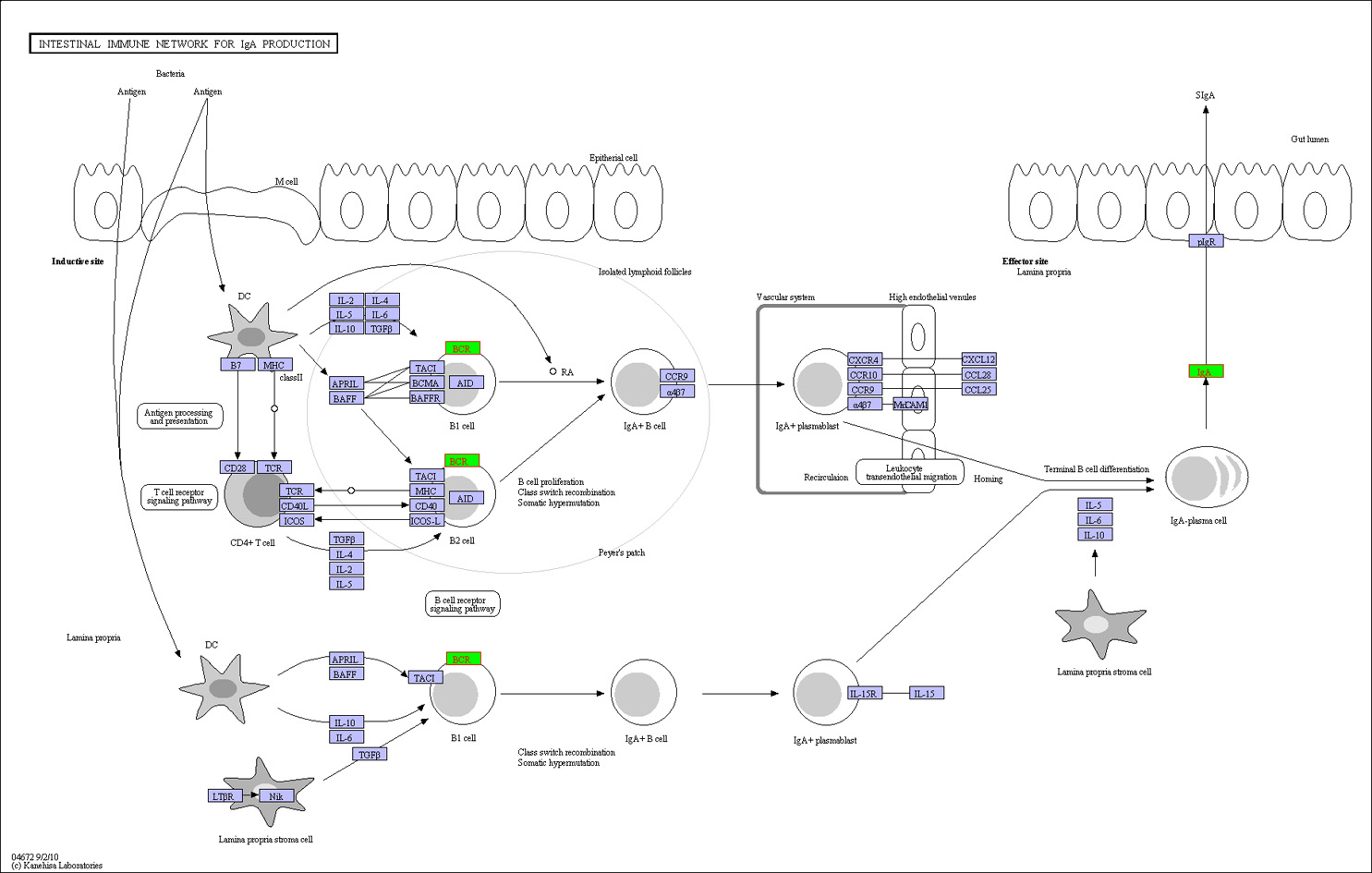

Supplement: S6 File — (ZIP) [file pone.0151597.s006.zip › S file 6/S4-15.jpg]

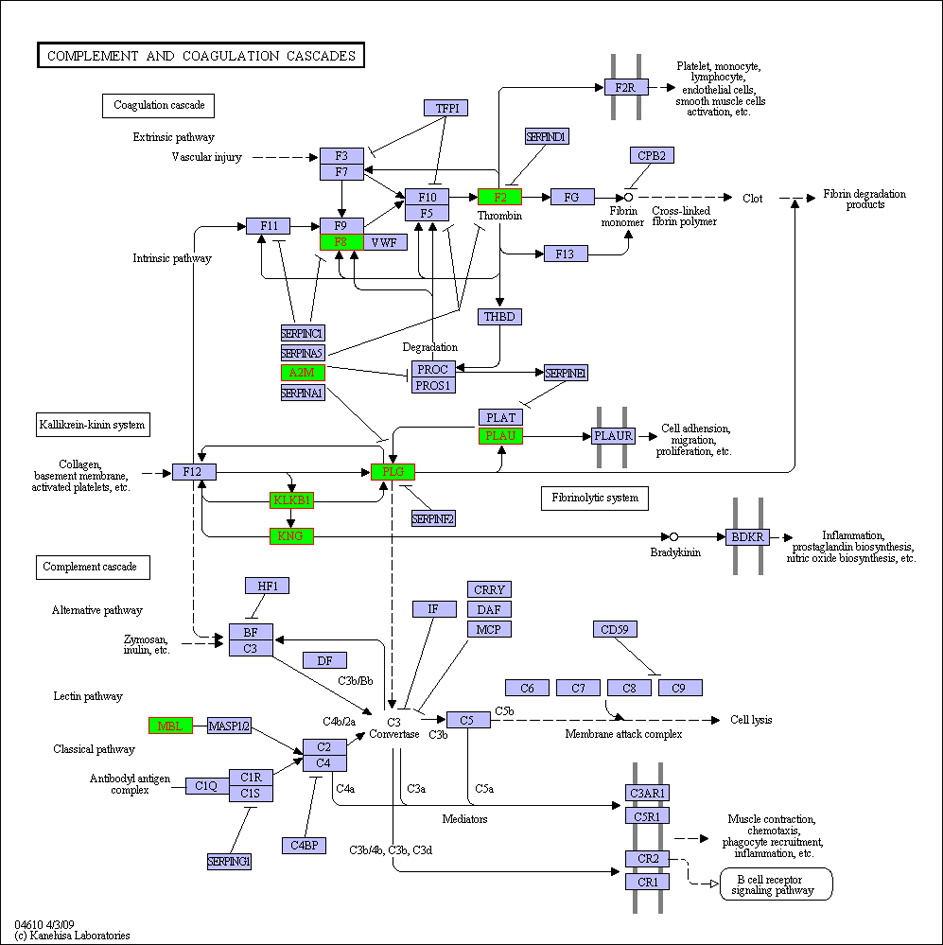

Supplement: S6 File — (ZIP) [file pone.0151597.s006.zip › S file 6/S4-2.jpg]

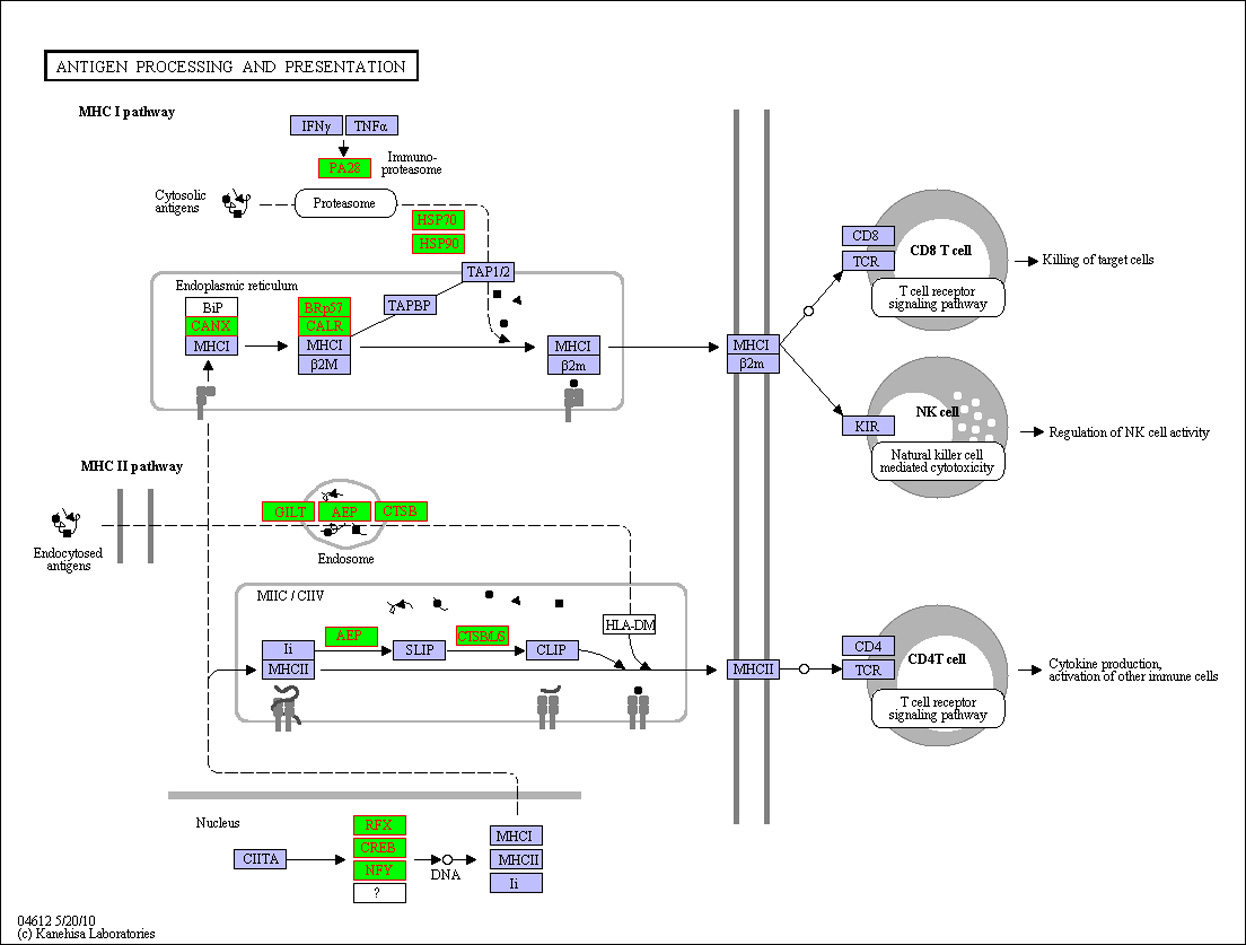

Supplement: S6 File — (ZIP) [file pone.0151597.s006.zip › S file 6/S4-3.jpg]

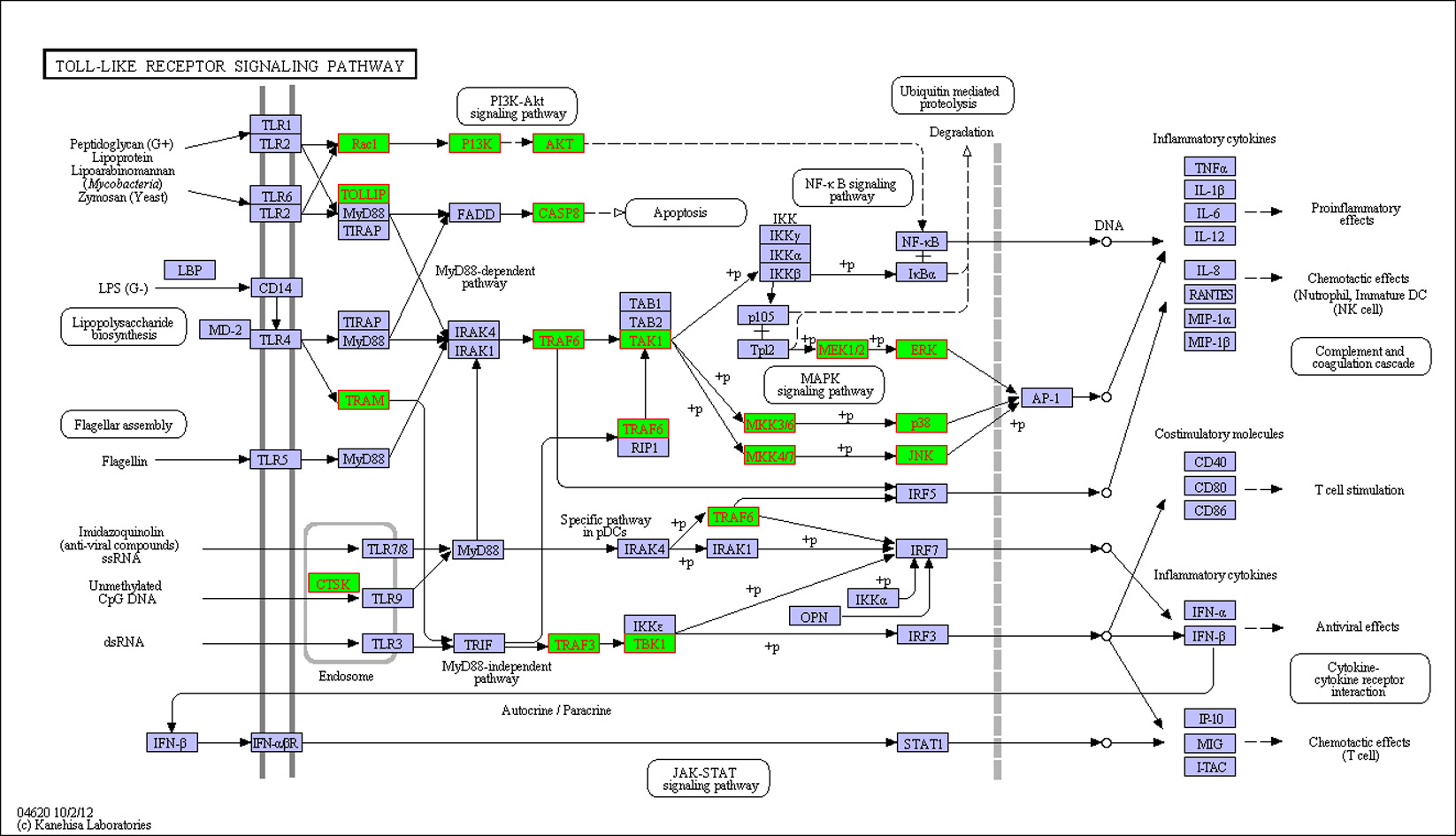

Supplement: S6 File — (ZIP) [file pone.0151597.s006.zip › S file 6/S4-4.jpg]

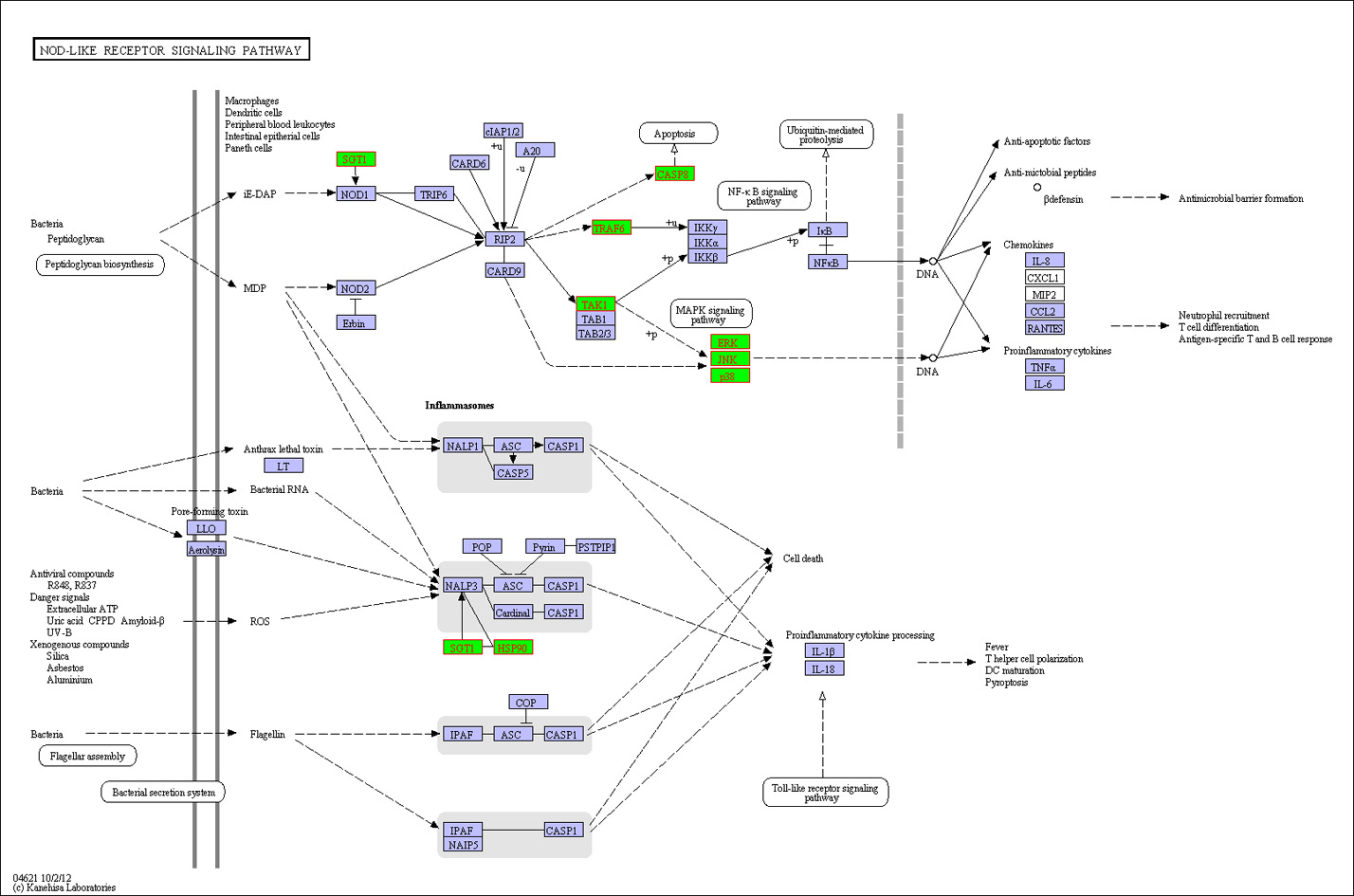

Supplement: S6 File — (ZIP) [file pone.0151597.s006.zip › S file 6/S4-5.jpg]

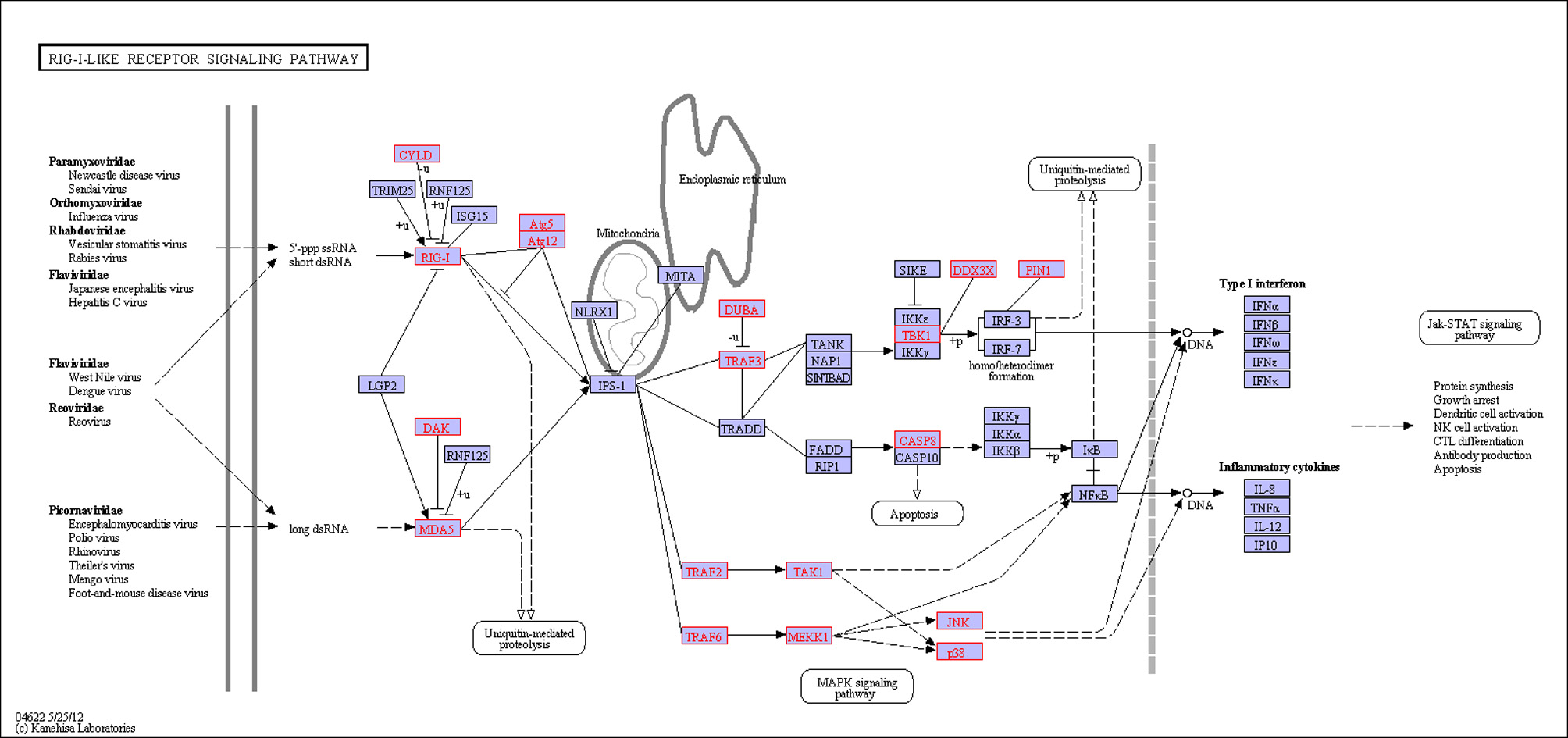

Supplement: S6 File — (ZIP) [file pone.0151597.s006.zip › S file 6/S4-6.jpg]

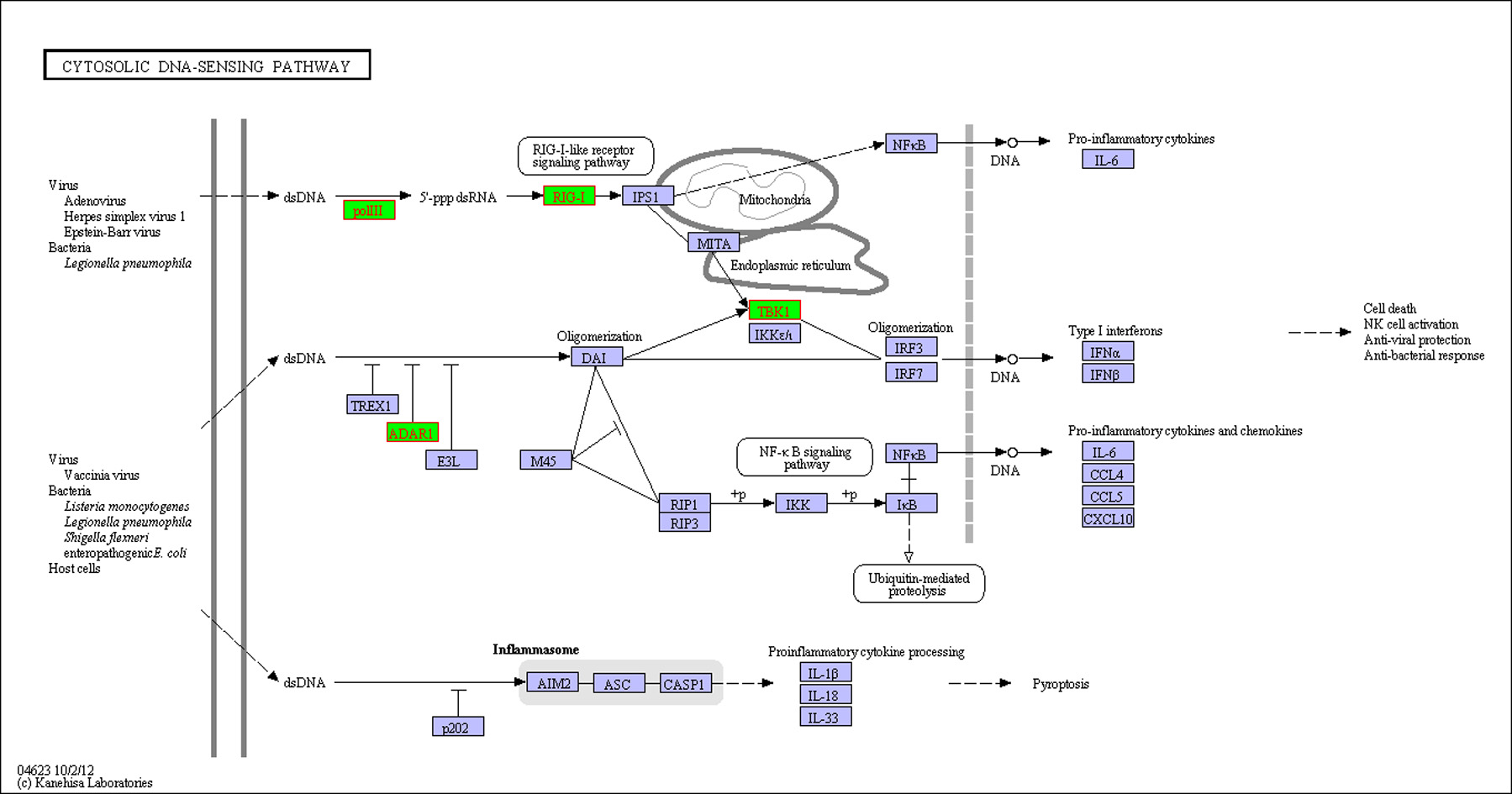

Supplement: S6 File — (ZIP) [file pone.0151597.s006.zip › S file 6/S4-7.jpg]

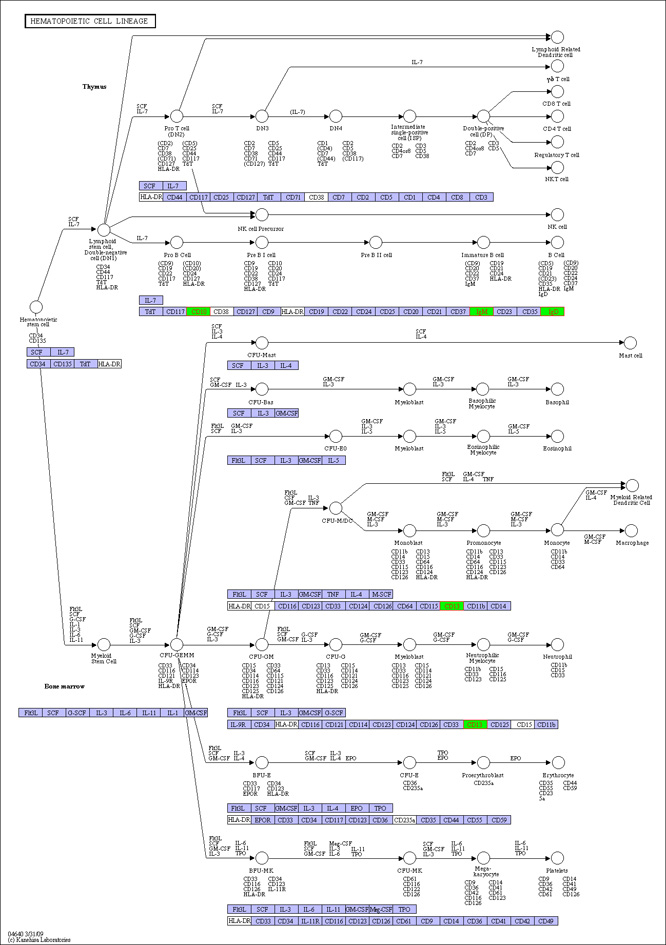

Supplement: S6 File — (ZIP) [file pone.0151597.s006.zip › S file 6/S4-8.jpg]

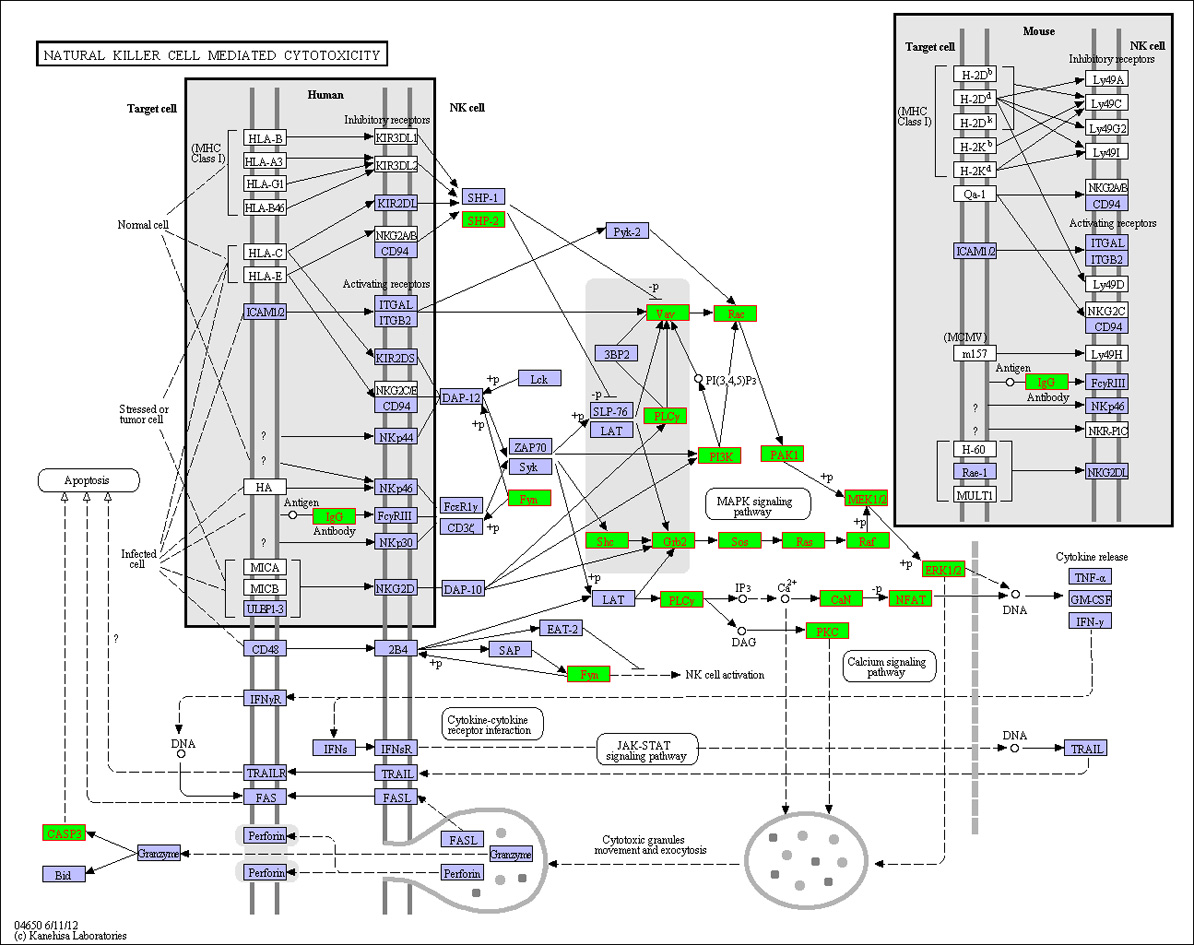

Supplement: S6 File — (ZIP) [file pone.0151597.s006.zip › S file 6/S4-9.jpg]

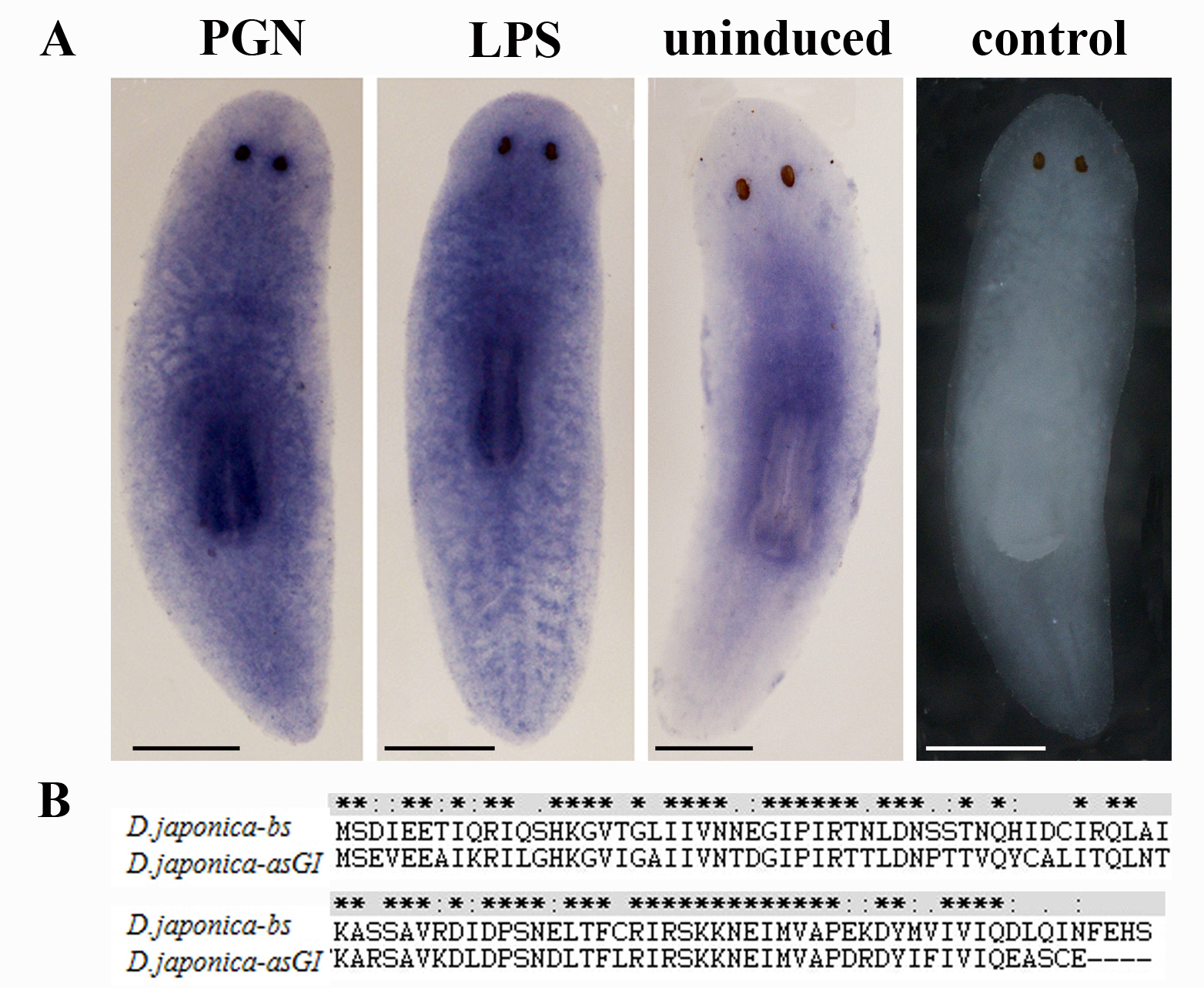

Supplement: S9 File — (TIF) [file pone.0151597.s009.tif]

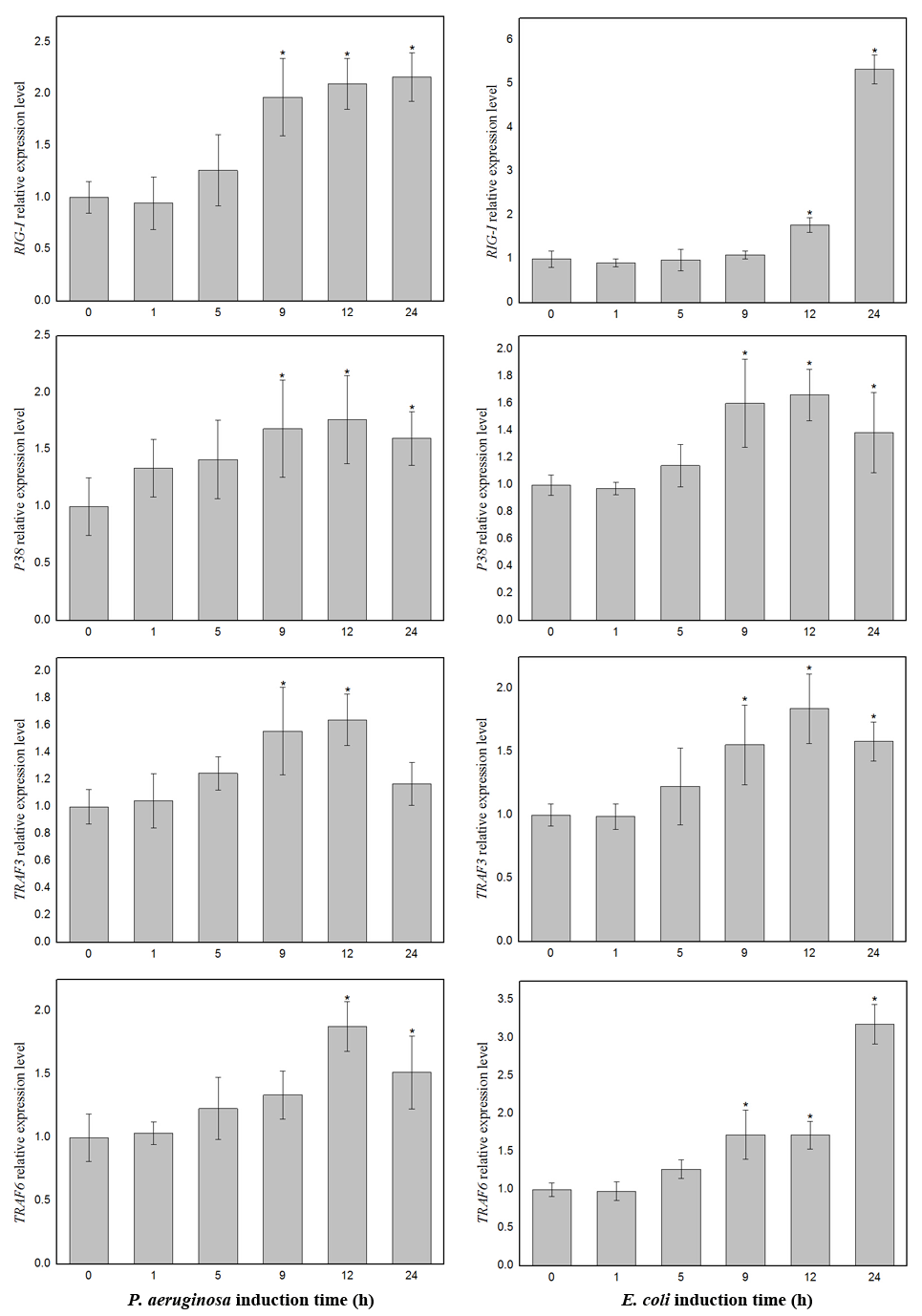

Supplement: S10 File — (ZIP) [file pone.0151597.s010.zip › S file 10/S10-2.tif]

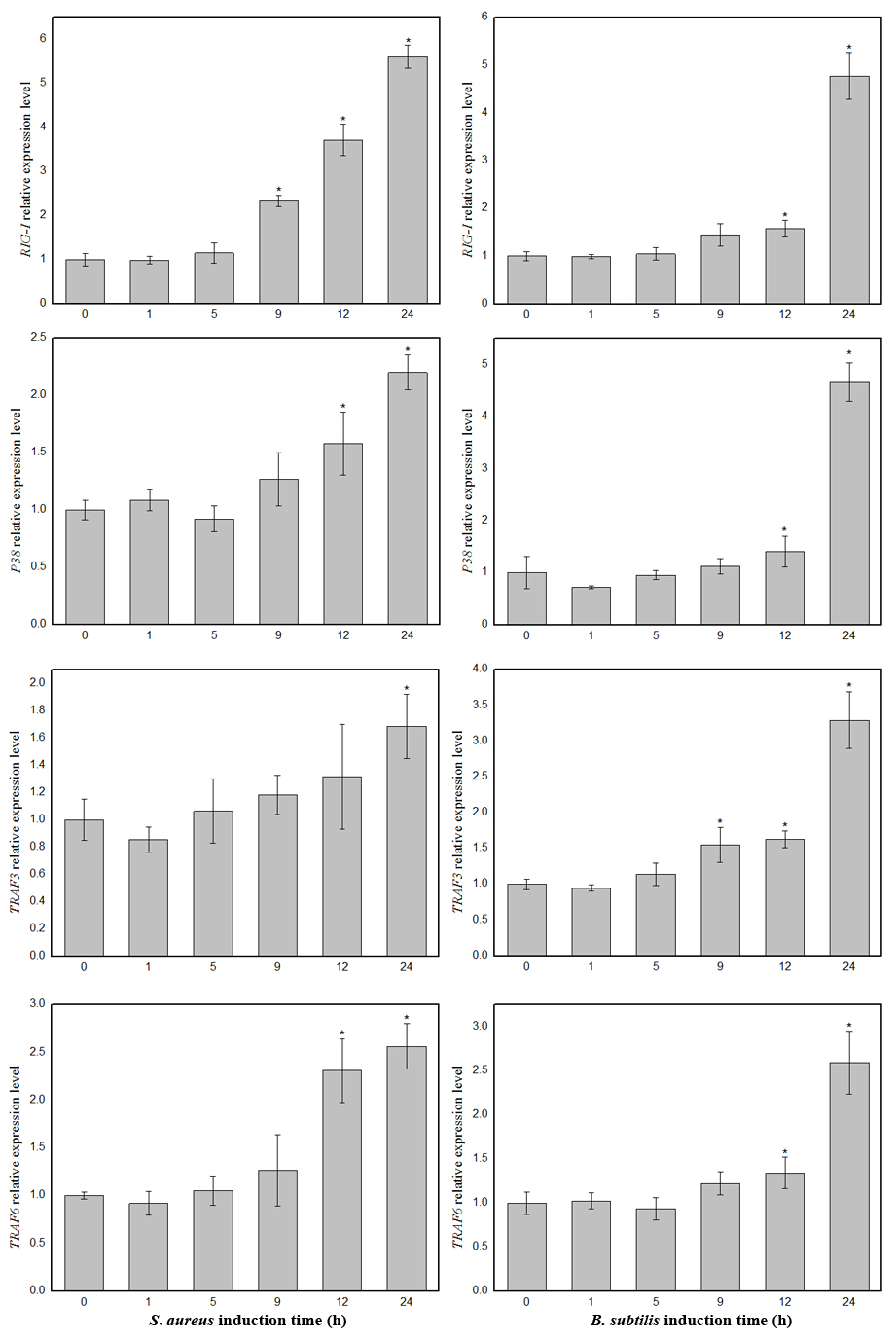

Supplement: S10 File — (ZIP) [file pone.0151597.s010.zip › S file 10/S10-3.tif]
